# Dual selections based knowledge transfer learning for cross-subject motor imagery EEG classification

**DOI:** 10.3389/fnins.2023.1274320

**Published:** 2023-11-28

**Authors:** Tian-jian Luo

**Affiliations:** ^1^College of Computer and Cyber Security, Fujian Normal University, Fuzhou, China; ^2^Digital Fujian Internet-of-Thing Laboratory of Environmental Monitoring, Fujian Normal University, Fuzhou, China

**Keywords:** motor imagery, electroencephalograph, cross-subject, feature selection, domain adaptation, noninvasive brain computer interface

## Abstract

**Introduction:**

Motor imagery electroencephalograph (MI-EEG) has attracted great attention in constructing non-invasive brain-computer interfaces (BCIs) due to its low-cost and convenience. However, only a few MI-EEG classification methods have been recently been applied to BCIs, mainly because they suffered from sample variability across subjects. To address this issue, the cross-subject scenario based on domain adaptation has been widely investigated. However, existing methods often encounter problems such as redundant features and incorrect pseudo-label predictions in the target domain.

**Methods:**

To achieve high performance cross-subject MI-EEG classification, this paper proposes a novel method called Dual Selections based Knowledge Transfer Learning (DS-KTL). DS-KTL selects both discriminative features from the source domain and corrects pseudo-labels from the target domain. The DS-KTL method applies centroid alignment to the samples initially, and then adopts Riemannian tangent space features for feature adaptation. During feature adaptation, dual selections are performed with regularizations, which enhance the classification performance during iterations.

**Results and discussion:**

Empirical studies conducted on two benchmark MI-EEG datasets demonstrate the feasibility and effectiveness of the proposed method under multi-source to single-target and single-source to single-target cross-subject strategies. The DS-KTL method achieves significant classification performance improvement with similar efficiency compared to state-of-the-art methods. Ablation studies are also conducted to evaluate the characteristics and parameters of the proposed DS-KTL method.

## 1 Introduction

Electroencephalograph (EEG) based noninvasive brain-computer interfaces (BCIs) play a significant role in neuroscience and cognitive science (Lebedev, 2014; Ramadan and Vasilakos, 2017; Abiri et al., 2019). Common EEG-BCI paradigms include event-related potential (ERP) (Wang et al., 2023b), steady-state visual evoked potential (SSVEP) (Schielke and Krekelberg, 2022), and motor imagery (MI) (Brusini et al., 2021), which involve signal processing and pattern recognition of EEG signals recorded during these paradigms. ERP and SSVEP belong to passive stimulus paradigms, which can often cause visual fatigue and have limited practical applications (Zhang et al., 2020a). In contrast to the aforementioned paradigms, the MI paradigm adopts a subject-driven approach, where subjects imagine self-generated limb movements across various scenarios and time durations. Therefore, it finds wide applications in areas such as rehabilitation assistance and brain-controlled games. During the process of MI, rhythmic modulation occurs in the sensorimotor cortical regions of the brain (Al-Saegh et al., 2021), commonly referred to as sensorimotor cortical regions. This modulation is reflected in the MI-EEG signals as power variations in specific frequency bands (Tangwiriyasakul et al., 2013). Typically, frequency band power variations occur in the contralateral sensorimotor cortical regions. For example, left-hand MI leads to power suppression in the right sensorimotor cortical regions, known as the event-related desynchronization (ERD) phenomenon (Nam et al., 2011).

The phenomenon of ERD primarily manifests as a decrease in power in the μ and β rhythms of MI-EEG signals (Ono et al., 2013). Therefore, it is crucial to analyze the power variations in these two rhythms of MI-EEG signals, extract appropriate features, and classify patterns corresponding to the MI task for building MI-BCI applications (Kevric and Subasi, 2017). Since EEG signals are multivariate time series, spatial domain features of MI-EEG signals are typically extracted for classification. Commonly used spatial feature representation methods based on the covariance matrix include common spatial patterns (CSP) (Ang et al., 2012) and Riemannian tangent space (RTS) (Xu et al., 2021). Classical methods for classifying spatial features include linear discriminant analysis (LDA). However, due to the nonlinear and non-stationary characteristics of EEG signals, the underlying MI patterns embedded in multivariate time series vary across different subjects and recording sessions (Wu et al., 2022). Furthermore, the inconvenience of EEG signal acquisition often makes it challenging to collect a sufficient number of samples from individual subjects to train high-performance classifiers. Additionally, the temporally varying and spatially coupled characteristics of MI-EEG signals across subjects make it difficult to directly use samples from different subjects to construct classifiers (Khademi et al., 2023). Therefore, the flexible individual-specific characteristics pose a significant challenge in the application of MI-EEG signals. For instance, in practical MI-BCI systems, labeled samples can only be obtained from existing subjects, and it is not possible to access a sufficient number of samples from newly arrived subjects.

To address this challenge, numerous transfer learning or domain adaptation based methods (Kouw and Loog, 2019) have been proposed to reduce the distribution divergence across subjects and enable cross-subject MI-EEG classification. Existing cross-subject MI-EEG classification methods can be generally divided into three categories based on the approach used for adaptation: sample alignment-based methods (Zanini et al., 2017; He and Wu, 2019; Zhang et al., 2021), feature adaptation based-methods (Zhang and Wu, 2020a,b; Cai et al., 2022; Jiang et al., 2022; Luo, 2022), and deep learning model-based methods (Zhao et al., 2020; Hong et al., 2021; She et al., 2023). Sample alignment-based methods aim to align the average covariance matrix of MI-EEG samples from each subject to an identity matrix, thereby bringing the distribution of multivariate time series closer. Feature adaptation-based methods draw inspiration from the field of cross-domain image transfer learning, where features are projected into the reproducing kernel Hilbert space (RKHS), and kernel methods are employed to learn domain-invariant features. The methods based on deep learning models primarily utilize convolutional neural network (CNN) to extract features from MI-EEG samples and incorporate feature alignment or feature adversarial techniques during gradient descent to achieve the learning of domain-invariant features. This ensures the performance of cross-subject MI-EEG classification.

Formally, deep learning-based methods require high computational resources and may not be suitable for constructing practical MI-BCI applications (Altaheri et al., 2023). Therefore, this study focuses on the first two categories of methods. Currently, the majority of cross-subject MI-EEG classification methods employ sample alignment as a preprocessing step. After aligning the MI-EEG samples, subjects are designated as the source domain and target domain, and the CSP or RTS spatial features are extracted separately. Subsequently, a feature adaptation strategy is developed for the spatial features, and the transformed domain-invariant features are used for classification. In existing feature adaptation methods, the primary consideration is given to the marginal and conditional distributions of the source and target domain features. To construct the conditional distribution of the unlabeled target domain, the model trained on the source domain is typically employed to predict pseudo-labels for the target domain (Long et al., 2013). Additionally, to ensure the stability of feature distribution during the iterative adaptation process, regularization terms are often incorporated into the optimization objective, ensuring the morphologies of features for each class (Zhang et al., 2017). Common regularization techniques include using the within-class scatter matrix to guarantee the discriminability of the source domain, utilizing the Laplacian matrix to preserve the locality of the target domain, and adopting the *l*_2_-norm to regularize the transformation matrix during the feature adaptation process.

Although present feature adaptation methods can achieve promising performance in cross-subject MI-EEG classification, we have observed that they still suffer from two drawbacks. First, most of them heavily rely on the predicted pseudo-labels of the target domain. Unfortunately, during the initial stages of domain shift, the MI-EEG samples from the target domain could be wrongly predicted, making it difficult to obtain an ideal conditional distribution for adaptation. Secondly, the spatial features deployed during the adaptation process may contain redundant components, which can significantly impact the performance and efficiency of feature adaptation. Thus, currently available methods lack consideration for abnormal feature dimensions and wrongly labeled samples in the target domain, which hampers the performance of cross-subject MI-EEG classification. In addition, the spatial feature of RTS suffers from extremely large dimensions when the number of EEG recording channels is large, further deteriorating the efficiency of iterative amendment of pseudo-labels in the target domain.

Currently, in the development of online BCIs, using existing subject datasets to construct cross-subject classification, there are two main research branches: conventional feature adaptation methods and adversarial deep learning models. The former focuses on achieving domain-invariant feature representation across different subjects, while the latter focuses on learning models capable of extracting domain-invariant features. Each branch has its own advantages and limitations. The conventional feature adaptation methods primarily emphasize target optimization, offering higher execution efficiency and lower cost requirements. However, their performance is constrained by the limitations of feature representation. On the other hand, adversarial deep learning models prioritize model training, and with an increasing number of subjects, they exhibit higher performance. Nevertheless, they are hindered by higher computational and storage costs, making them less suitable for application in low-power wearable EEG devices. To address the goal of constructing an online BCI on wearable devices with limited storage capacity, this study focuses on conventional feature adaptation methods. By employing superior feature selection and representation techniques, as well as pseudo-label selection, the purpose is to achieve higher performance in terms of BCI classification.

To meet the aforementioned requirements, we propose DS-KTL (short for Dual Selections based Knowledge Transfer Learning), a novel solution that integrates supervised feature selection and pseudo-label selection into the manifold regularized feature adaptation. DS-KTL method aims to improve the performance of cross-subject MI-EEG classification. Specifically, DS-KTL iteratively obtains accurately pseudo-labels of the unlabeled target domain under the selected discriminant feature dimensions across the source domain and target domain.

The contributions of this work are summarized as follow:

1. Two steps of manifold embedded spatial feature selection and pseudo-label selection with incremental confidence are introduced for both source domain and target domain during feature adaptation, resulting in excellent performance in cross-subject MI-EEG classification.
2. A dual selections has been introduced to the manifold regularized feature adaptation framework, which further reduces the distribution discrepancy across subjects, and automatically regularizes the abnormal feature dimensions and pseudo-labels.
3. The proposed method is free from the dimensional explosion problem, and the dimensions of the manifold embedded spatial features can be tuned during real-world applications. Meanwhile, extensive experiments on various benchmark datasets are conducted to demonstrate the efficacy of the proposed method.

The rest of this paper is organized as follows. Section 2 gives a brief review of the most related work. In Section 3, we present the details of the proposed method. Section 4 provides a series of experimental results, and the discussion is given in Section 5. In Section 6, we provide a conclusion of this paper.

## 2 Related works

In this section, we give a brief review of existing cross-subject MI-EEG classification methods which can be generally grouped into three categories.

### 2.1 Sample alignment methods

The idea of sample alignment of MI-EEG signals for the cross-subject classification is derived from the domain generalization (Zhou et al., 2022), which adjusts the sample distributions of all subjects to a similar distribution. Since the statistical information of MI-EEG samples is embedded in covariance matrices, researchers have considered covariance matrix as the sample distribution and performed alignment on the average covariance matrix of all samples. The first method to align covariance matrices on the Riemannian space was Riemannian alignment (RA) (Zanini et al., 2017). RA aligns the covariance matrices from all subjects to the average covariance of an identity matrix, thereby reducing the distribution discrepancy among subjects. The aligned covariance matrices can then be classified using the minimum distance mean classifier. However, due to the high time complexity of the Riemannian centroid computation, researchers proposed the Euclidean alignment (EA) method (He and Wu, 2019) to align samples based on the Euclidean average of covariance matrices, which significantly improve the computation efficiency. Instead of constructing the classifier on the covariance matrices, EA aligns MI-EEG samples while preserving their multivariate time-series form. To further enhance MI-EEG classification, researchers extract spatial features such as CSP or RTS from the aligned samples, providing flexible for classification. More recently, researchers have built upon RA and EA by extracting CSP features from sub-bands, revising the alignment objective, and performing target alignment on these features (SB-TA-CSP) (Zhang et al., 2021). This approach has shown improved performance in cross-subject MI-EEG classification.

### 2.2 Feature adaptation methods

The idea of feature alignment of CSP or RTS spatial features for cross-subject MI-EEG classification is derived from the domain adaptation. The first method introduced for aligning the marginal distribution in cross-domain image classification is transfer component analysis. Researchers aimed to align both the marginal and conditional distributions in cross-domain image classification and proposed methods such as joint distribution alignment (JDA) (Long et al., 2013), joint probability distribution alignment (JPDA) (Zhang and Wu, 2020a), and balanced distribution alignment (BDA) (Wang et al., 2017) based on pseudo-labels from the target domain. Recently, iterative distribution alignment methods were developed by adding regularizations, such as joint geometrical and statistical alignment (JGSA) (Zhang et al., 2017) and selective pseudo-labeling (SPL) (Wang and Breckon, 2020). Other researchers focused on subspace alignment and proposed methods like correlation alignment (CROAL) (Sun et al., 2017). These domain adaptation methods based on CSP or RTS spatial features utilized kernel tricks during distribution alignment. Furthermore, researchers have also developed the domain adaptation methods specifically for cross-subject MI-EEG classification, including manifold embedded knowledge transfer (MEKT) (Zhang and Wu, 2020b), manifold embedded transfer learning (METL) (Cai et al., 2022), kernel based manifold domain adaptation (KMDA) (Jiang et al., 2022), and feature weighting regularized joint probability distribution adaptation (FWR-JPDA) (Luo, 2022). These methods adapt the original CSP or RTS spatial features through complex optimizations. However, the pseudo-labels from the target domain often introduce errors during iterative distribution alignment process, which can deteriorate the performance of cross-subject MI-EEG classification.

Generally, the CSP or RTS features are effective in decoupling MI-EEG samples, and their efficient computation process and lower feature dimension make them suitable for conditional distribution alignment. Moreover, obtaining pseudo-labels iteratively in conditional distribution alignment allows for the continuous correction of incorrect labels in the target domain, enabling the source domain-trained classifier to generalize effectively to the target domain. Additionally, methods such as MEKT, METL, KMDA, and FWR-JPDA incorporate regularization techniques, such as manifold transformations, or feature structure preservation to enhance generalization during feature adaptation. However, mainstream feature adaptation methods rely on RTS or CSP features, which include redundant features that are considered together during the feature adaptation process, posing challenges in domain adaptation. On the other hand, although pseudo-labels based conditional distribution alignment improves generalization, it becomes challenging to effectively align the conditional distribution if a large number of erroneous pseudo-labels are generated from the initial iterations, especially for challenging MI-EEG samples. In summary, the performance of feature adaptation is determined by the feature representation in the source domain and the acquisition of pseudo-labels in the target domain, which are the two key points our proposed method aims to address.

### 2.3 Deep learning models

Domain adversarial methods represent another branch of cross-subject MI-EEG classification based on deep learning models (Chen et al., 2022). Initially applied to cross-domain image classification, researchers empolyed domain adversarial neural network (DANN) (Li et al., 2018) for this purpose. Recently, deep learning models inspired by the DANN have been utilized in cross-subject MI-EEG classification. One such model is the deep representation based domain adaptation (DRDA) model (Zhao et al., 2020), which optimizes three modules simultaneously: feature extractor, classifier, and domain discriminator. By obtaining domain-invariant deep representations, the DRDA model enhances MI-EEG classification performance. To address differences in sub-domain distributions among different MI classes, which the DRDA model overlooks, researchers introduced the dynamic joint DANN (DJDAN) model (Hong et al., 2021). The DJDAN model incorporates multiple sub-domain discriminators for domain adversarial learning. Furthermore, to improve the performance of domain adversarial learning, researchers introduced the Wasserstein distance to the DRDAN model (She et al., 2023), resulting in significant performance improvements in cross-subject MI-EEG classification. However, adversarial learning based on deep neural networks requires substantial training time to achieve subject-invariant features extracting models, which is impractical in real-world MI-BCI scenarios. Therefore, our proposed method focuses on sample alignment and feature adaptation, enabling rapid adaptation to newly arrived subjects and facilitating real-time online applications of MI-BCI systems.

## 3 The proposed method

In this section, we first provide the preprocessing steps for sample alignment and spatial feature extraction. Then, we introduce the method for selecting manifold embedded spatial features. Next, we briefly introduce the widely used joint probability distribution adaptation method and the manifold regularization approach with pseudo-labels selection during selected spatial feature adaption. Finally, we give the overview of the proposed method and analyze its computational complexity.

### 3.1 Preliminaries

Based on transfer learning or domain adaptation (Kouw and Loog, 2019), the formal mathematical definition of cross-subject MI-EEG classification can be depicted as: Given a set of MI-EEG signals extracted from *m* subjects, represented as $D_{m}={\left\{x_{i},y_{{\;}_{i}}\right\}}_{i=1}^{n}$, where each subject contains *n* samples. Among them, each EEG sample is denoted as *x* ∈ *R*^ch*Ts^, where *ch* represents the number of EEG recording channels, *Ts* represents the number of sampling points, and *y* represents the label of the corresponding MI task. The objective is to select one subject with unlabeled samples as the target domain $D_{t}={\left\{x_{i}\right\}}_{i=1}^{n_{t}}$, and select partial or one subject with labeled samples as the source domain $D_{s}={\left\{x_{i},y_{{\;}_{i}}\right\}}_{i=1}^{n_{s}}$. It is assumed that the feature space is *X*_*s*_ = *X*_*t*_, and label space is *L*_*s*_ = *L*_*t*_, but it contains the marginal probability *P*_*s*_(*x*_*s*_) ≠ *P*_*t*_(*x*_*t*_), and the conditional probability *P*_*s*_(*y*_*s*_|*x*_*s*_) ≠ *P*_*t*_(*y*_*t*_|*x*_*t*_). The goal is to perform the transfer learning or domain adaptation method to train a classifier on the source domain to predict labels on the target domain with minimal loss. Table 1 illustrates the frequently used symbols and notations in the paper.

**Table 1:** Frequently used symbols and notations in the paper.

**Symbol**	**Description**	**Symbol**	**Description**
*D*_*m*_	Dataset of *m* subjects	*n*	Number of samples of each subject
*D*_*s*_	Dataset of source domain	$\bar{C}$	Mean of covariance matrix
*D*_*t*_	Dataset of target domain	*M*	Riemannian covariance matrix
*x*_*i*_	The *i*-th MI-EEG sample	*f*_*s*_	Feature set of source domain
${\hat{x}}_{i}$	Aligned *i*-th MI-EEG sample	*f*_*t*_	Feature set of target domain
*y*_*i*_	Label of *i*-th MI-EEG sample	*c*/*C*	Class index of each MI task
*v*	Embedded transformed vector	*I*	Identity matrix
*p*	Manifold embedded projection	α, β, γ	Parameters of feature selection
*J*	Degree matrix	*K*	Manifold embedded graph
*V*	Auxiliary matrix to solve ℓ_2,1_	$f_{s}^{*}$	Selected feature of source domain
*L*	Laplacian matrix	$f_{t}^{*}$	Selected feature of target domain
ϕ_*T*_	Transferability of adaptation	λ	Parameter of adaptation
ϕ_*D*_	Discriminability of adaptation	ω_*s*_	Optimal source adaptation vector
ξ(·)	Feature adaptation function	ω_*t*_	Optimal target adaptation vector
*r*_*s*_	Constructed source label matrix	*r*_*t*_	Constructed target pseudo label matrix
*P*_*s*_/*P*_*t*_	Normalized source /target label vector	*Q*_*s*_/*Q*_*t*_	Normalized source /target label matrix
*S*_*w*_	Within-class scatter matrix	*S*_*b*_	Between-class scatter matrix
μ, η, σ	Parameters of regularizations	ρ	Lagrangian multiplier
*H*	Centering matrix for adaptation	ℓ	Loss function
$u_{s}^{*}$	Nearest source class prototype	$u_{t}^{*}$	Optimal target class prototype

### 3.2 Centroid alignment and spatial feature extraction

Recently, RA and EA based centroid alignment (CA) of covariance matrix for the raw MI-EEG samples from each subject has been recommended by the researchers (Zhang and Wu, 2020b; Luo, 2022) to be a preprocessing step to narrow the distribution discrepancy across subjects, since the covariance represents the distribution characteristics of MI-EEG samples. Therefore, the first step is to perform the centroid alignment. Firstly, the centroid is represented by the arithmetic average of the *m*th subject:


(1) $$\begin{array}{c} \bar{C}=\frac{1}{n}\overset{n}{\underset{i=1}{\sum }}x_{i}x_{i}^{\text{T}} \end{array}$$

Then, the alignment procedure regards as a reference matrix to align each MI-EEG sample from the *m*th subject:


(2) $$\begin{array}{c} {\hat{x}}_{i}={\bar{C}}^{-\frac{1}{2}}x_{i} \end{array}$$

For each subject from *D*_*m*_, a same alignment procedure is performed by Equations 1 and 2. Finally, after alignment, we can also compute the centroid of covariance for the *m*th subject:


(3) $$\begin{array}{c} {\bar{C}}^{\prime }\text{=}\frac{1}{n}\overset{n}{\underset{i=1}{\sum }}{\hat{x}}_{i}{\hat{x}}_{i}^{\text{T}}=\frac{1}{n}\overset{n}{\underset{i=1}{\sum }}{\bar{C}}^{-\frac{1}{2}}x_{i}x_{i}^{\text{T}}{\bar{C}}^{-\frac{1}{2}}={\bar{C}}^{-\frac{1}{2}}\bar{C}{\bar{C}}^{-\frac{1}{2}}=I \end{array}$$

The result points out that the centroid of covariance for each subject corresponds to the identity matrix, which represents a similar samples distribution across *m* subjects. The centroid of covariance, computed by Equation 2, is based on Euclidean mean and can be easily extended to the log-Euclidean mean by adding the log(·) operation. Furthermore, the covariance of MI-EEG samples is a symmetric positive definite (SPD) matrix, which can be viewed as a differential Riemannian manifold (Zanini et al., 2017). Hence, the Riemannian mean can also be computed to represent the centroid. Before introducing the Riemannian mean, the Riemannian distance between two SPD matrices *M*_1_ and *M*_2_ can be defined as:


(4) $$\begin{array}{c} d\left(M_{1},M_{2}\right)=\|\log\left(M_{1}^{-1}M_{2}\right)|{|}_{F} \end{array}$$

where ‖•‖_*F*_ denotes the Frobenius norm, and log(·) is the logarithm form of eigenvalues that computed from $M_{1}^{-1}M_{2}$. Based on Riemannian distance, the Riemannian mean of *n* samples can be computed as:


(5) $$\begin{array}{c} mean_{M}={\arg\min}_{M}\overset{n}{\underset{i=1}{\sum }}d\left(M,M_{i}\right) \end{array}$$

The way in which the centroid is computed can influence the alignment of MI-EEG samples and lead to varying performance across different datasets and transfer strategies. Therefore, during experiment, we will show the performance using different mean computing of centroid alignment, and present excellent results.

Typically, we extract the spatial features from the aligned MI-EEG samples for subsequent feature adaptation. The CSP and RTS are two commonly used spatial features. Since CSP is a supervised method and its dimensionality is influenced by the number of channels, we select to use the RTS feature for the following feature adaptation. Formally, the RTS feature projects the Riemannian SPD matrix *M*_*i*_ onto the tangent space surrounding the Riemannian SPD *M*, and obtains the Euclidean tangent space vector, which can be represented as:


(6) $$\begin{array}{c} f=upper\left(\log_{M}\left(M_{ref}M_{i}M_{ref}\right)\right) \end{array}$$

where *upper*(•) is upper triangular part extraction operator to obtain the Euclidean tangent space vector $f_{i}\in R^{1*\frac{ch\left(ch+1\right)}{2}}$ for *M*_*i*_. For the datasets used in the experiment, Dataset 2a contains *ch* = 22 that will produce 253-dimensional RTS features, and Dataset 2b contains *ch* = 3 that will produce 6-dimensional RTS features. During feature extraction, the reference matrix can be defined as $M_{ref}=M^{-\frac{1}{2}}$ to confirm the homomorphism of the extracted Euclidean tangent space vector from the Riemannian manifold.

### 3.3 Manifold embedded spatial feature selection

Researchers (Yan et al., 2021) have highlighted that the presence of redundant features can hinder the feature adaptation process. Therefore, prior to feature adaptation, we propose using a supervised manifold embedded feature selection (MEFS) method to select discriminative features for the following feature adaptation. Our feature selection method is inspired by the approach described in reference (Zhang et al., 2019), which aims to construct a linear transformation of the original feature space to approximate a low-dimensional embedded feature space. Since the labeled samples are from the source domain, we utilize these samples for feature selection, and select the same feature dimensions for the unlabeled samples from the target domain. Given the labeled samples of the RTS spatial feature vector ${\left\{f_{i},y_{{\;}_{i}}\right\}}_{i=1}^{n_{s}}\in D_{s}$, and a low-dimensional embedded projection *p* with the transformed feature vector ν, the MEFS method aims to learn the linear transformation *fν* − *p*.

To ensure the supervised process, the labels *y* should be incorporated as a regularization term in the projection *p* − *y*. Additionally, the Laplacian regularization term *Tr*(*p*^T^*Lp*) is employed to preserve the structural properties of the embedded features on the manifold. To perform the feature selection within the linear approximation, the *l*_2,1_-norm is introduced to automatically select representative features in the low-dimensional manifold embedding. Ultimately, the objective of the MEFS can be formulated as:


(7) $$\begin{array}{c} \min_{p,\nu }\;\|f\nu -p{\|}_{F}^{2}+\alpha Tr\left(p^{\text{T}}Lp\right)+\beta \|p-y{\|}_{F}^{2}+\gamma \|\nu |{|}_{2,1} \end{array}$$

where *L* = *J* − *K* is the Laplacian matrix of graph *J*. Graph *J* is selected to be computed by the heat kernel, and *K* represents a degree matrix (Belkin and Niyogi, 2004). The parameters α, β serve as counterbalance parameters between the two regularizations, while γ is the parameter associated with the *l*_2,1_-norm. It is important to note that all parameters are fine-tuned in the experiments conducted on different MI-EEG datasets.

Since the *l*_2,1_-norm is non-smooth, the objective of the MEFS needs to be solved by an alternating minimization strategy. First, we fixed the transformed feature vector ν, and then compute the derivative of Equation 7 with respect to (w.r.t) *p* as follow:


(8) $$\begin{array}{c} \left(\alpha L+\left(\beta +1\right)I\right)p=f\nu +\beta y \end{array}$$

where *L, I, f*, ν, *y* are known, so the form of *AX* + *XB* = *C*(*B* = ϕ) w.r.t Equation 8 can be easily solved using the Matlab function lyap, and the analytical solution of the low-dimensional embedded projection $\tilde{p}$ minimized can be secured. Next, we fixed the secured *p*, and the derivative of Equation 7 w.r.t ν can be computed as:


(9) $$\begin{array}{c} \min_{\nu }\|f\nu -p{\|}_{F}^{2}+\gamma \|\nu |{|}_{2,1} \end{array}$$

Since the Equation 9 is non-convex w.r.t the *l*_2,1_-norm, a commonly used method is to introduce an auxiliary matrix $V=\left[\begin{array}{ccc} \frac{1}{2{\left\|\nu_{1}\right\|}_{2}} & 0 & 0\\ 0 & ... & 0\\ 0 & 0 & \frac{1}{2{\left\|\nu_{\frac{ch\left(ch+1\right)}{2}}\right\|}_{2}} \end{array}\right]$, and revisited Equation 9 to:


(10) $$\begin{array}{c} \nu ={\left(f^{\text{T}}f+\gamma V\right)}^{-1}f^{\text{T}}p \end{array}$$

In the alternating minimization strategy, the aforementioned solutions w.r.t ≠ and *p* are iterated alternately until reaching the preset maximum iterations or achieving minimum errors. For the realized sparse feature vector, the representative of each dimension is measured based on a supervised way. Hence, for any ∀*i*, the top-*q* feature dimensions $f_{i}^{s}\in R^{1*q}$ are selected as the most discriminant features for the subsequent feature adaptation. Regarding the RTS features of the source domain and target domain, the selected RTS features can be represented as $f_{s}^{*}={\left\{f_{i}^{*},y_{{\;}_{i}}\right\}}_{i=1}^{n_{s}}\in D_{s},f_{t}^{*}={\left\{f_{i}^{*}\right\}}_{i=1}^{n_{t}}\in D_{t}$.

### 3.4 Joint probability distribution adaptation

To facilitate the adaptation of the selected RTS features between the source domain and target domain, the joint probability distribution adaptation (JPDA) (Zhang and Wu, 2020a) is commenced, which is widely used for feature adaptation in the context of the MI-EEG signals (Zhang and Wu, 2020b; Luo, 2022). The JPDA method is derived from the TCA and JDA, which confirms the intra-class transferability and discriminability during adaptation. For the selected RTS features of $f_{s}^{*}$ and $f_{t}^{*}$, the transferability across domains and the discriminability across classes ϕ_*D*_ should be considered during the adaptation ξ(·):


(11) $$\begin{array}{c} \xi \left(f_{s}^{*},f_{t}^{*}\right)=\phi_{T}-\lambda \phi_{D} \end{array}$$

where λ is the trade-off parameter between two terms. Due to the consideration of intra-class discriminability, we assume that for two classes of MI, the transferability ϕ_*T*_ can be defined based on the transformation vector ω = [ω_*s*_; ω_*t*_]:


(12) $$\begin{array}{c} \phi_{T}=\overset{2}{\underset{c=1}{\sum }}{\left\|\frac{1}{n_{s}^{c}}\overset{n_{s}^{c}}{\underset{i=1}{\sum }}\omega_{s}^{\text{T}}f_{s,i}^{*,c}-\frac{1}{n_{t}^{c}}\overset{n_{t}^{c}}{\underset{j=1}{\sum }}\omega_{t}^{\text{T}}f_{t,j}^{*,c}\right\|}_{F}^{2} \end{array}$$

where $n_{s}^{c}$ and $n_{t}^{c}$ are the number of samples of class *c* for the corresponding domains. The purpose of transferability ϕ_*T*_ is to project the original feature vector to an embedded space based on ω = [ω_*s*_; ω_*t*_]. Since the solver of JPDA needs the pseudo-labels of the target domain, we follow the one-hot label matrix coding of the JPDA method, and define the label matrix of source domain and target domain as *y*_*s*_ = [*y*_*s*,1_, ..., *y*_*s*,_*n*__*s*__] and ỹ_*t*_ = [ỹ_*t*,1_, ..., ỹ_*t*,_*n*__*t*__] for the transferability ϕ_*T*_.

Also, the discriminability ϕ_*D*_ can be clarified as:


(13) $$\begin{array}{c} \phi_{D}=\underset{c\neq c^{\prime }}{\sum }\overset{2}{\underset{c^{\prime }=1}{\sum }}{\left\|\frac{1}{n_{s}^{c}}\overset{n_{s}^{c}}{\underset{i=1}{\sum }}\omega_{s}^{\text{T}}f_{s,i}^{*,c}-\frac{1}{n_{t}^{c^{\prime }}}\overset{n_{t}^{c^{\prime }}}{\underset{j=1}{\sum }}\omega_{t}^{\text{T}}f_{t,j}^{*,c^{\prime }}\right\|}_{F}^{2} \end{array}$$

where *c*′ represents the class that different from *c*, and the purpose of discriminability ϕ_*D*_ is to maximize the discriminability among different classes. Similarly, we respectively defined the label matrices for the discriminability for both source domain and target domain. Specifically, the *r*_*s*_ represents the combination of *c*(*c* = 1, 2)-th column of the source labels, and we repeat 1 times to construct label matrices, while ${\hat{r}}_{t}$ represents the 1 times combination from the 1-th column to the 2-th column of pseudo labels from the target domain. Notably, the *r*_*s*_ is fixed from the source domain labels *y*_*s*_ during feature adaptation, while the ${\hat{r}}_{t}$ is iteratively updated during the pseudo labels ŷ_*t*_ selected from the target domain.

To give a formally form of the transferability and discriminability based on the maximum mean discrepancy (MMD) (Chen et al., 2019), the objective of Equation 11 can be depicted as:


(14) $$\begin{array}{c} \min_{\omega_{s},\omega_{t}}\|\omega_{s}^{\text{T}}f_{s}^{*}P_{s}-\omega_{t}^{\text{T}}f_{t}^{*}P_{t}{\|}_{F}^{2}-\lambda \|\omega_{s}^{\text{T}}f_{s}^{*}Q_{s}-\omega_{t}^{\text{T}}f_{t}^{*}Q_{t}{\|}_{F}^{2} \end{array}$$

where *P*_*s*_ = *y*_*s*_/*n*_*s*_, *P*_*t*_ = ŷ_*t*_/*n*_*t*_ and $Q_{s}=r_{s}/n_{s},Q_{t}={\hat{r}}_{t}/n_{t}$ represent the normalized one-hot label matrix.

### 3.5 Regularizations of feature adaptation

The JPDA method of Equation 14 is deprivation of regularizations, which will cause overfitting during feature adaptation. Followed by the regularizations of MEKT (Zhang and Wu, 2020b) and JGSA (Zhang et al., 2017), we also added three regularizations for the feature adaptation.

#### 3.5.1 Discriminability of the source domain

Commonly, since the features from the source domain are labeled, the discriminability of the source domain can be preserved by the within-class and between-class scatter matrix:


(15) $$\begin{array}{c} \begin{array}{c} \min_{\omega_{s}}\;Tr\left(\omega_{s}^{\text{T}}S_{w}\omega_{s}\right)\\ s.t.\;\omega_{s}^{\text{T}}S_{b}\omega_{s}=I \end{array} \end{array}$$

Among them, the within-class scatter matrix *S*_*w*_ is defined as:


(16) $$\begin{array}{c} S_{w}=\sum_{c=1}^{2}\sum_{i=1}^{n_{s}^{c}}\left(1-1/n_{s}^{c}\right)(f_{s,i}^{*,c}{)}^{\text{T}}f_{s,i}^{*,c} \end{array}$$

And the between-class scatter matrix is *S*_*b*_ defined as:


(17) $$\begin{array}{c} S_{b}=\sum_{c=1}^{2}n_{s}\left(f_{s,m}^{*,c}-f_{s,m}^{*}\right){\left(f_{s,m}^{*,c}-f_{s,m}^{*}\right)}^{\text{T}} \end{array}$$

where $f_{s,m}^{*,c}$ is the mean of features from class *c*, and $f_{s,m}^{*}$ is the mean of features from all classes.

#### 3.5.2 Locality preservation of the target domain

However, the features from the target domain are non-labeled, so we only launch the Laplacian regularization to preserve the locality of the target domain like the MEFS process. It can be given as:


(18) $$\begin{array}{c} \begin{array}{c} \min_{\omega_{t}}\;Tr\left(\omega_{t}^{\text{T}}f_{t}^{*}L{\left(f_{t}^{*}\right)}^{\text{T}}\omega_{t}\right)\\ s.t.\;\omega_{t}^{\text{T}}f_{t}^{*}H{\left(f_{t}^{*}\right)}^{\text{T}}\omega_{t}=I \end{array} \end{array}$$

where *L* is the normalized form from Equation 7, which can be defined as *L* = *I* − *J*^−1/2^*KJ*^−1/2^. Moreover, the centering matrix $H=I-\frac{1}{n_{t}}1_{n_{t}},1_{n_{t}}\in R^{n_{t}*n_{t}}$ is used to limit the scaling effect during feature adaptation (Belkin and Niyogi, 2003).

#### 3.5.3 Regularization of the transformation vector

The similarity of the transformation vectors ω = [ω_*s*_; ω_*t*_] respect to the source domain and target domain should be regularized for a better generalization performance. To alleviate the extreme values of such two transformation vectors, the regularziation term of ω_*s*_ and ω_*t*_ should be added:


(19) $$\begin{array}{c} \begin{array}{c} \min_{\omega_{s},\omega_{t}}\;\left(\|\omega_{t}-\omega_{s}{\|}_{F}^{2}+\|\omega_{t}{\|}_{F}^{2}\right) \end{array} \end{array}$$

By adding the three regularizations, the Equation 14 can be rewritten to:


(20) $$\begin{array}{c} \begin{array}{c} \min_{\omega_{s},\omega_{t}}\;\|\omega_{s}^{\text{T}}f_{s}^{*}P_{s}-\omega_{t}^{\text{T}}f_{t}^{*}P_{t}{\|}_{F}^{2}-\lambda \|\omega_{s}^{\text{T}}f_{s}^{*}Q_{s}-\omega_{t}^{\text{T}}f_{t}^{*}Q_{t}{\|}_{F}^{2}+\\ \eta Tr\left(\omega_{s}^{\text{T}}S_{w}\omega_{s}\right)+\mu Tr\left(\omega_{t}^{\text{T}}f_{t}^{*}L{\left(f_{t}^{*}\right)}^{\text{T}}\omega_{t}\right)+\sigma \left(\|\omega_{t}-\omega_{s}{\|}_{F}^{2}+\|\omega_{t}{\|}_{F}^{2}\right)\\ s.t.\;\omega_{s}^{\text{T}}S_{b}\omega_{s}=I,\omega_{t}^{\text{T}}f_{t}^{*}H{\left(f_{t}^{*}\right)}^{\text{T}}\omega_{t}=I \end{array} \end{array}$$

where η, μ, σ are the counterbalance parameters across the three regularizations, which are fine-tuned in the experiments for different MI-EEG datasets.

### 3.6 Pseudo labels selection of feature adaptation

The solver of Equation 20 can be easily performed due to it is convex. By introducing the Lagrangian multiplier method to the s.t. conditions, the solver of Equation 20 is represented as:


(21) $$\begin{array}{c} \ell =Tr(\omega^{\text{T}}(\left[f_{s}^{*};f_{t}^{*}\right]\left(A_{\min}-\lambda A_{\max}\text{+}\eta B+\sigma C\right){\left[f_{s}^{*};f_{t}^{*}\right]}^{\text{T}}\\ \;+\mu D)\omega +\rho \left(I-\omega^{\text{T}}E\omega )\right) \end{array}$$

where $A_{\min}=\left[\begin{array}{cc} P_{s}P_{s}^{T} & -P_{s}P_{t}^{T}\\ -P_{t}P_{s}^{T} & P_{t}P_{t}^{T} \end{array}\right],A_{\max}=\left[\begin{array}{cc} Q_{s}Q_{s}^{T} & -Q_{s}Q_{t}^{T}\\ -Q_{t}Q_{s}^{T} & Q_{t}Q_{t}^{T} \end{array}\right],B=\left[\begin{array}{cc} S_{w} & 0\\ 0 & 0 \end{array}\right],C=\left[\begin{array}{cc} 0 & 0\\ 0 & L \end{array}\right],D=\left[\begin{array}{cc} I & -I\\ -I & 2I \end{array}\right]$, and ρ is the parameter of Lagrangian operator with $E=\left[\begin{array}{cc} S_{b} & 0\\ 0 & \left[f_{s}^{*};f_{t}^{*}\right]H{\left[f_{s}^{*};f_{t}^{*}\right]}^{\text{T}} \end{array}\right]$. By setting the derivative of $\frac{\partial \ell }{\partial \omega }=0$, the solution is:


(22) $$\begin{array}{c} \left(\left[f_{s}^{*};f_{t}^{*}\right]\left(A_{\min}-\lambda A_{\max}\text{+}\eta B+\sigma C\right){\left[f_{s}^{*};f_{t}^{*}\right]}^{\text{T}}+\mu D\right)\omega =\rho E\omega \end{array}$$

Equation 22 can be easily solved using the Matlab function eigs, and the transformation matrix ω is obtained from the *z* trailing eigenvectors, where *z* represents the dimension of embedded feature space. For the original feature adaptation, the solver of Equation 22 is computed iteratively, as the matrix should be constructed based on the pseudo-labels of the target domain. To obtain effective pseudo-labels, it is common practice to train an LDA/shrinkage LDA classifier on the transformed source domain features $u_{s}^{*}=\omega^{\text{T}}f_{s}^{*}$, and then use this classifier to generate pseudo-labels for the transformed target domain features $u_{t}^{*}=\omega^{\text{T}}f_{t}^{*}$. Through iterative refinement, the accuracy of the pseudo-labels for the target domain gradually improves over time.

Unfortunately, the nonlinear and non-stationary characteristics of MI-EEG samples often lead to initial incorrect pseudo-label predictions by the classifier trained on source domain during the early iterations. This issue significantly affects the feature adaptation process. Previous studies (Wang and Breckon, 2020; Teng et al., 2022) have shown that gradually predicting pseudo-labels from high-quality to low-quality samples yields better results than directly predicting pseudo labels for all samples from the target domain during the feature adaptation process. Therefore, to ensure a well-performing iterative process of feature adaptation, we employ a selective pseudo-labeling strategy. This strategy involves selectively predicting pseudo-labels for target domain samples during the iterative process. As the the classifier's confidence increases, more and more target domain samples are correctly predicted.

To measure the confidence of the classifier, we first define the nearest class prototype on the samples from the source domain:


(23) $$\begin{array}{c} {ū}_{s}^{y}=\frac{\sum_{i=1}^{n_{s}}u_{s}^{*}Iden\left(y,y_{s,i}\right)}{\sum_{i=1}^{n_{s}}Iden\left(y,y_{s,i}\right)},Iden\left(y,y_{i}\right)=\{\begin{array}{c} 1,y=y_{i}\\ 0,otherwise \end{array} \end{array}$$

Based on the nearest class prototype, the conditional probability of the samples from the source domain can be computed as:


(24) $$\begin{array}{c} P\left(c|x_{t}\right)=\frac{e^{-\|u_{t}^{*}-{ū}_{s}^{y}\|}}{\sum_{c=1}^{2}e^{-\|u_{t}^{*}-{ū}_{s}^{y}\|}} \end{array}$$

Instead of using the LDA/shrinkage LDA classifier, based on the conditional probability, the pseudo-labels of the target domain can be easily obtained:


(25) $$\begin{array}{c} {ỹ}_{t}=\arg\max\left\{P\left(c|x_{t}\right)\right\} \end{array}$$

Based on the pseudo labels for all samples ỹ_*t*_ from the target domain, and the corresponding conditional probability *P*(ỹ_*t*_|*x*_*t*_), the dataset of the target domain of the first iteration can be defined as ${\tilde{D}}_{t}={\left\{{ỹ}_{i},x_{i},P\left({ỹ}_{i}|x_{i}\right)\right\}}_{i=1}^{n_{t}}$. During iteration of feature adaptation, we select a group of high confidence (high probability) samples from the target domain to achieve the pseudo-labels. The number of selected pseudo-labels is related within the number of iterations *T*. For the *i*th iteration, the class-wise top $m_{t}=i*n_{t}^{c}/T$ samples with the highest probability are selected from the target domain for the next iteration. It is important to note that the top selected pseudo-labeled samples are unified from each class, thereby avoiding the risk of exclusively selecting samples from the specific classes. Consequently, in the subsequent iterations of feature adaptation, the $f_{t}^{*}$ in solver Equation 25 is replaced by the selected *m*_*t*_ samples. It is worth noting that the nearest class prototype conditional probability classifier is defined as a binary classifier by Equation 23 to 25. For a scenario of *n* classes of MI-EEG samples, we need to divide them into n*(*n* − 1)/2 binary classification tasks.

### 3.7 Overview of the proposed method

**Figure 1:**
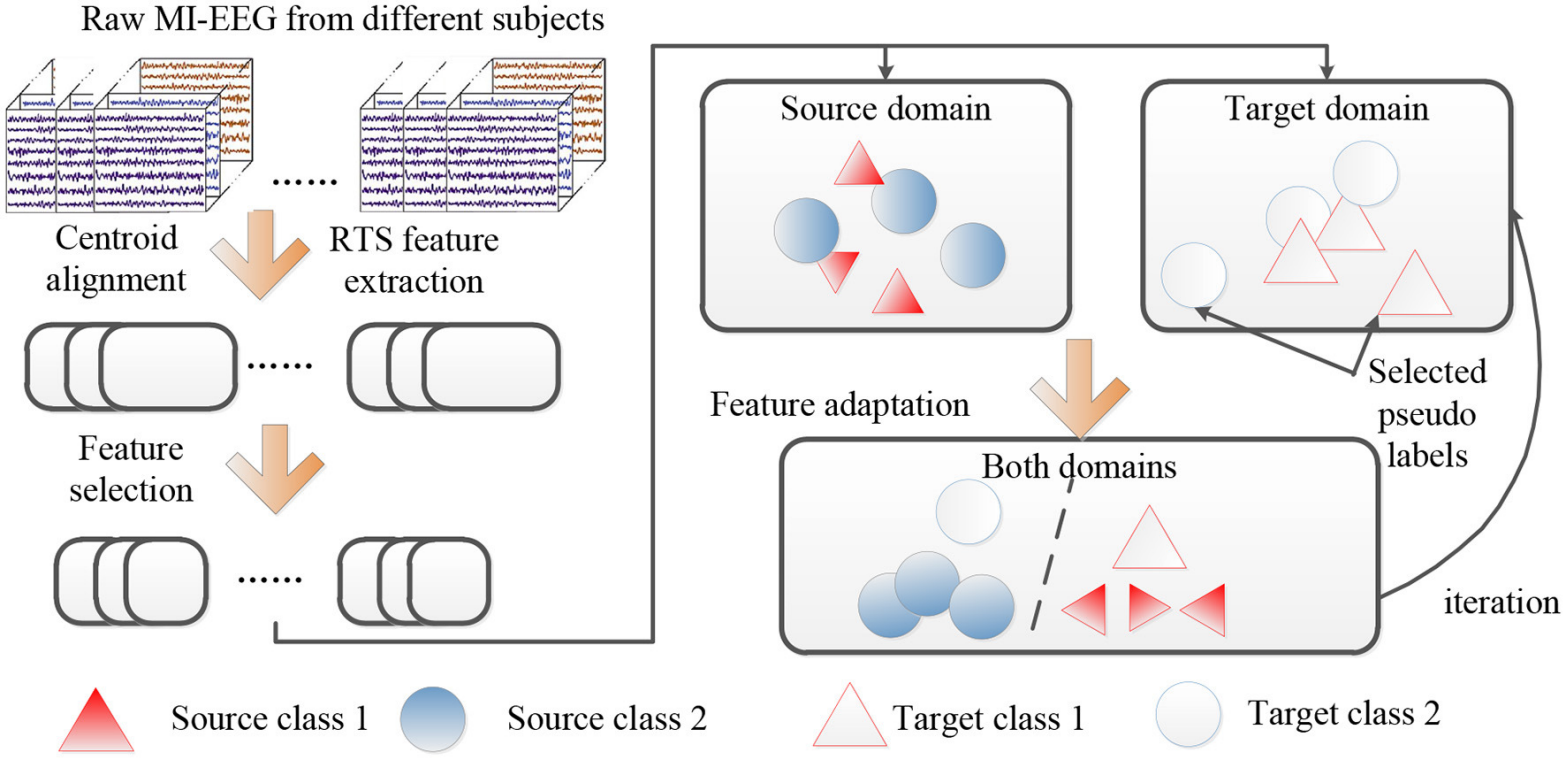
The flow chart of the proposed DS-KTL method.

Based on the aforementioned descriptions, we provide a concise overview of the proposed method, and the flow chart of the proposed DS-KTL method is illustrated in Figure 1. Furthermore, Algorithm 1 gives the pseudo-code of the proposed DS-KTL method for cross-subject MI-EEG classification. The proposed DS-KTL method consists of four steps: covariance alignment, feature extraction, feature selection, and feature adaptation with pseudo-label selection. Considering a total of *n* = *n*_*s*_ + *n*_*t*_ samples for the cross-subject EEG classification, we utilize the big-*O* notation to analyze the computational complexity.

**Algorithm 1:** DS-KTL for cross-subject MI-EEG classification.

**Input:** MI-EEG signals dataset $D_{m}={\left\{x_{i},y_{{\;}_{i}}\right\}}_{i=1}^{n}$ with *m* subjects;
**Output:** The results of each subject as the target domain ${\tilde{D}}_{t}={\left\{{ỹ}_{i},x_{i},P\left({ỹ}_{i}|x_{i}\right)\right\}}_{i=1}^{n_{t}}$.
1: Setting parameters α, β, γ, *q* for feature selection and λ, η, μ, σ, *z, T* for feature adaptation;
2: Compute the arithmetic average of covariance matrices by Equation 1;
3: Align MI-EEG samples for each subject by Equation 2;
4: Extract spatial features for each subject by Equation 6;
5: Select discriminative features for each subject by solve Equation 7;
6: for ***k* = 1, 2, ..., *m* do**
7: MTS: Treat each subject *D*_*k*_ as the target domain and the rest *m* − 1 subjects as the source domain;
8: STS: Treat each subject *D*_*k*_ as the target domain and another subject $D_{k}^{\prime }$ as the source domain;
9: According to each MTS/STS cross-subject MI-EEG classification task, construct *A*_min_, *A*_max_,
10: *B, C, D* for Equation 22;
11: **for *i* = 1, 2, ..., *T* do**
12: Solve Equation 22, and construct ω as the *z* trailing eigenvectors;
13: Compute the transformed features of source domain $u_{s}^{*}$ and target domain $u_{t}^{*}$;
14: Compute the nearest class prototype of source domain ${ū}_{s}^{y}$ by Equation 23;
15: Compute the conditional probability of the target domain *P*(*c*|*x*_*t*_) by Equation 24,
16: and the corresponding pseudo-labels ỹ_*t*_;
17: Select the class-wise top $m_{t}=i*n_{t}^{c}/T$, and update $f_{t}^{*}$ in Equation 22;
18: **end**
19: **end**
20: return ỹ_*t*_, *P*(*c*|*x*_*t*_)

Firstly, the covariance alignment consumes a time complexity of *O*(*n*^2^) to compute the mean of covariance. Then, the feature extraction requires a time complexity of *O*(n*ch^2^) with respect to the number of channels in MI-EEG signals. For the feature selection, a time complexity of *O*(*n*^2^) is used to select a feature dimension of *D*. During the feature adaptation for a total of *T* iterations, the number of $n_{t}^{\prime }$ samples have been selected as the pseudo-labels, so the MMD is constructed by $O\left({\left(n_{s}+n_{t}^{\prime }\right)}^{2}\right)$, and the regularizations of transferable and discriminability has a time complexity of $O\left(D\left(n_{s}+n_{t}^{\prime }\right)\right)$, as well as the three regularizations. Finally, the eigen-decomposition of Equation 22 takes a time complexity of *O*(*D*^2^), and the nearest class prototype requires a time complexity of *O*(2*D*) for the binary MI tasks. In conclusion, the total theoretical computational complexity is:


(26) $$\begin{array}{c} O\left(n^{2}+n*ch^{2}+T\left({\left(n_{s}+n_{t}^{\prime }\right)}^{2}+3D\left(n_{s}+n_{t}^{\prime }\right)+D^{2}+2D\right)\right) \end{array}$$

We can observe that the proposed DS-KTL method exhibits a comparable time complexity when compared to the state-of-the-art methods. The empirical efficiency will be further compared and discussed in the experiments.

## 4 Experiments and results

### 4.1 Datasets

**Figure 2:**
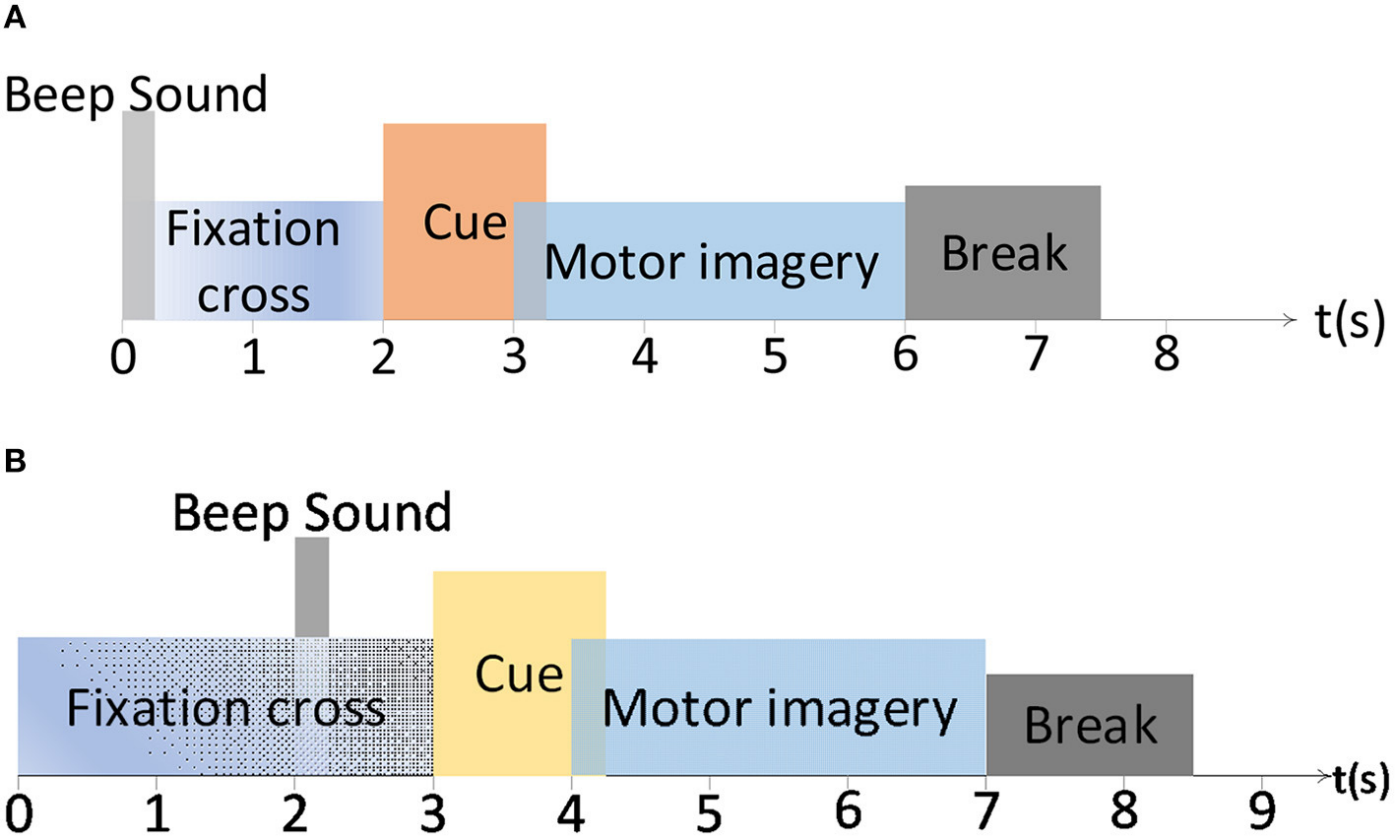
The paradigm of the motor imagery for BCIIV-2a and BCIIV-2b datasets. **(A)** BCIIV-2a dataset. **(B)** BCIIV-2b dataset.

Two widely used public benchmark MI-EEG datasets have been introduced to evaluate the proposed DS-KTL method. Figure 2 exhibits the experimental paradigm for the BCIIV-2a and BCIIV-2b datasets. These two selected datasets shared a similar MI paradigm, except for the different MI onset moment. The experiment commenced with the appearance of a cross at the center of the screen with a beep sound, which aimed to capture the subjects attention. Afterwards, a cue of MI task prompt was presented to the subject, and the subjects engaged in a 4-s MI process based on the provided prompts. Following the completion of each MI task, a rest period of 1.5 s was given before proceeding to the next task. The details of the MI-EEG datasets are as follow (Tangermann et al., 2012):

(1) BCI Competition IV dataset 2a (BCIIV-2a): Four MI tasks of left hand (L), right hand (R), feet (F), and tongue (T) prompts were presented to nine healthy subjects. During MI paradigm, an EEG recording device with 22 electrodes and 250 Hz sampling rate was applied for data collection. Two sessions, training and testing, have been conducted with each session collect 288 samples. We selected the training session for cross-subject MI-EEG classification, and each class contained 72 samples. We primarily conducted MI-EEG classification experiments for (L vs. R) and (R vs. T) tasks. Additionally, for the 4 MI tasks (L, R, F, and T) in this dataset, we also provided experimental results on 4*(4 − 1)/2 = 6 binary classification tasks: Task 1 (L vs. R), Task 2 (L vs. F), Task 3 (L vs. T), Task 4 (R vs. F), Task 5 (R vs. T), and Task 6 (F vs. T). Due to article space limitations, the ablation study results are only provided for Task 1 and Task 6.

(2) BCI Competition IV dataset 2b (BCIIV-2b): This dataset also collects the MI-EEG signals from nine healthy subjects, including left hand (L) and right hand (R) prompts. The experimental setup consisted of 3 EEG electrodes, and the sampling rate was set at 250 Hz. The data collection process was divided into five sessions. The first two sessions contained 120 samples each, while the remaining three periods contained either 160 or 120 samples each. Due to the limited number of electrodes in the BCIIV-2b dataset, we selected the samples recorded during the first three sessions for the cross-subject MI-EEG classification experiment. This resulted in a total of 400 MI-EEG samples of each subject (200 for L class and 200 for R class), with any excess samples removed for some subjects.

Similarly, to ensure a fair comparison of the experimental results, we selected the time intervals of [2.5, 6s] and [3.5, 7s] respectively, for the BCIIV-2a and BCIIV-2b datasets. Each sample in the datasets had a time series consisting of 750 sampling points. Table 2 presents the statistical information of the two datasets used in the experiment.

**Table 2:** The statistical information of two datasets for experiments.

**Datasets**	**Subjects**	**Samples**	**Channels**	**Sampling points**	**Classes**
BCIIV-2a (L vs. R)	9	144	22	750	2
BCIIV-2a (F vs. T)	9	144	22	750	2
BCIIV-2b	9	400	3	750	2

### 4.2 Baseline methods

To validate the superiority of the proposed DS-KTL method, we compare it with eight state-of-the-art (SOTA) methods. These methods are recently proposed and popularly compared for the cross-subject MI-EEG classification:

1. RA-MDM (Zanini et al., 2017): This method introduces Riemannian alignment for the first time. It first computes the covariance matrix of MI-EEG samples from each subject in the Riemannian space and then aligns the Riemannian centroids to the identity matrix. To classify the aligned covariance matrices, the minimum distance of the Riemannian means (MDM) classifier is used, assigning the class of a covariance matrix to the class corresponding to the nearest Riemannian mean.
2. EA-CSP-LDA (He and Wu, 2019): This method introduces Euclidean alignment for the first time. It directly computes the Euclidean centroid of the covariance matrices of MI-EEG samples from each subject. Subsequently, the MI-EEG samples are aligned to the Euclidean centroid. To classify the MI-EEG samples, CSP features are extracted, and an LDA classifier is used for classification.
3. CA-CORAL (Sun et al., 2017): This method applies Euclidean-based centroid alignment to align the MI-EEG samples from each subject, followed by extracting CSP features from the aligned MI-EEG samples. Finally, the CSP feature sets are divided into source and target domains, and correlation alignment (COLAR) is used to align the two domains on subspaces with classification using an LDA classifier.
4. CA-JDA (Long et al., 2013): This method applies Euclidean-based centroid alignment to align the MI-EEG samples from each subject, followed by extracting CSP features from the aligned MI-EEG samples. To align the conditional distribution, the CSP feature set is divided into the source and target domains, and the joint distribution adaptation (JDA) method is applied. An LDA classifier is used to iteratively obtain pseudo-labels for the target domain.
5. MEKT (Zhang and Wu, 2020b): This method applies Euclidean-based centroid alignment to align the MI-EEG samples from each subject, followed by extracting RTS features from the aligned MI-EEG samples. In the feature adaptation process, the joint probability distribution adaptation method is employed. The effective feature structure of the source and target domains is preserved iteratively through within-class and between-class scatter matrices, and pseudo-labels for the target domain are obtained using a shrinkage LDA classifier.
6. METL (Cai et al., 2022): This method applies Euclidean-based centroid alignment to align the MI-EEG samples from each subject, followed by extracting RTS features from the aligned MI-EEG samples. In the feature adaptation process, the joint probability distribution adaptation method is employed. The effective feature structure of the source and target domains is preserved iteratively through within-class and between-class scatter matrices, and pseudo-labels for the target domain are obtained using a shrinkage LDA classifier.
7. SB-TA-CSP (Zhang et al., 2021): This method first applies multiple sub-band filters to perform band-pass filtering on the MI-EEG samples from each subject. The source and target domains are then divided, and the samples from the source domain are aligned to the target domain for each filtering band. After extracting CSP features, feature selection is performed using minimum redundancy maximum relevance. Finally, an LDA classifier is used to iteratively obtain pseudo-labels for the target domain.
8. FWR-JPDA (Luo, 2022): This method applies Euclidean-based centroid alignment to align the MI-EEG samples from each subject, followed by extracting CSP features from the aligned MI-EEG samples. To enhance the generalization of feature adaptation, feature weighting regularized CSP features are selected to construct the joint probability distribution adaptation process. An eigenfeature regularized and extracted classifier is used to iteratively obtain pseudo-labels for the target domain.

Note that hyper-parameters of all baselines were set according to the recommendations in their corresponding publications.

### 4.3 Experimental setups

Following the same settings of the references, we evaluated the proposed DS-KTL method on two cross-subject classification scenarios, single-source to single-target (STS), and multi-source to single-target (MTS), respectively. For the nine subjects included in the BCIIV-2a and BCIIV-2b datasets, the STS strategy involves selecting one subject as the source domain and another subject as the target domain for each cross-subject MI-EEG classification task. Consequently, the STS strategy generates a total of 9*8 = 72 sub-tasks. Conversely, the MTS strategy selects one subject as the target domain and the remaining eight subjects as the source domain for each task. This strategy generates a total of nine sub-tasks.

We adopt classification “Accuracy” on each test set as the evaluation metric, which is widely used in existing references (Zhang and Wu, 2020b; Luo, 2022):


(27) $$\begin{array}{c} Accuracy=\frac{\left|x:x\in D_{t}\wedge ŷ\left(x\right)=y\left(x\right)\right|}{\left|x:x\in D_{t}\right|} \end{array}$$

where *y*(*x*) and ŷ(*x*) represent the ground truth and predicted labels for the target domain, respectively. We reported the average classification accuracy for each subject under the STS or MTS strategy as the final result. Given the inclusion of various parameters in the DS-KTL method, we employed a trial-and-error strategy to search for the optimal parameter settings for each dataset under the STS and MTS strategies. The default settings of the parameters for feature selection were α = 1, β = 1, γ = 100, while the parameters for knowledge transfer learning were η = 0.01, μ = 0.1, σ = 20. Other optimal parameter settings are exhibited in Table 3, where *q* represents the selected number of spatial features, λ denotes the counterbalance of JPDA, *z* indicates the subspace dimension of JPDA, and *T* is the maximum number of iterations. Additionally, we will discuss the parameter selections of the proposed DS-KTL method during ablation study section.

**Table 3:** Optimal parameter settings of DS-KTL method.

**MTS/STS strategy**	***q***	**λ**	***z***	***T***
BCIIV-2a (L vs. R)	180/252	0.01/0.01	100/100	5/10
BCIIV-2a (F vs. T)	150/180	0.01/0.01	100/100	5/10
BCIIV-2b	5/5	0.1/0.1	10/10	5/10

### 4.4 Results

#### 4.4.1 Performance of cross-subject classification

Tables 4–6 illustrate the cross-subject classification results on BCIIV-2a (L vs. R), BCIIV-2a (F vs. T), and BCIIV-2b, respectively. It is important to note that since the MEFS method involves a non-convex optimization process, random results with slight differences may be obtained during convergence. Therefore, we performed ten times of the experiment and reported the best classification results. For the STS strategy, each subject was treated as the target domain, with the remaining each subject serving as the source domain. The average classification accuracy was reported. Based on the results in the tables, it can be determined that regardless of the STS or MTS strategy, the Euclidean mean achieves the highest average classification accuracy in centroid alignment for all three classification tasks in BCIIV-2a and BCIIV-2b. The average classification accuracy was higher compared to aligning the MI-EEG samples with Riemannian mean or log-Euclidean mean.

**Table 4:** Cross-subject classification results on BCIIV-2a (L vs. R).

**Subjects**	**STS-R**	**STS-LE**	**STS-E**	**MTS-R**	**MTS-LE**	**MTS-E**
S1	74.22	74.91	84.64	83.33	81.94	91.67
S2	53.47	53.65	52.60	54.17	53.47	55.56
S3	79.25	79.17	85.94	96.53	96.53	99.31
S4	64.93	64.67	66.23	72.22	75.00	78.47
S5	58.68	57.99	56.42	58.33	59.03	58.33
S6	63.80	63.63	63.89	66.06	66.67	70.14
S7	62.85	62.59	63.45	71.53	70.14	71.53
S8	88.02	88.37	92.80	93.75	92.36	95.14
S9	78.91	78.73	78.56	83.33	84.03	82.64
Average	69.35	69.30	**71.61**	75.47	75.46	**78.09**
Standard deviation	11.29	11.47	14.23	14.85	14.60	15.62

The approaches with the highest average accuracy are denoted as bold.

Furthermore, the standard deviation of the Euclidean mean is higher than that of the other two means, indicating greater variability in classification accuracy across different subjects. By examining the classification results for each subject, we can conclude that the Euclidean mean significantly improves the classification accuracy in cases with larger class separability. However, the improvement is not substantial for cases with smaller class separability, and in some subjects, there is a slight inhibitory effect. Therefore, selecting the appropriate mean calculation method for centroid alignment becomes crucial, depending on the distribution of different MI-EEG datasets.

Specifically, subjects that exhibit high classification accuracy as the target domain under the MTS strategy also tend to have high accuracy when used as the source domain under the STS strategy. For example, S1, S3, S8, and S9 in Table 4, S8 in Table 5, and S4 in Table 6. These subjects consistently achieve stable accuracy as both the source and target domains across different tasks and are often referred to as “golden subjects” (Sun et al., 2022). They are well-suited for classification applications in online MI-BCI scenarios. Similarly, some subjects that pose challenges for classification show unsatisfactory performance in both MTS and STS strategies, for instance, S2, S5, and S6 in Tables 4, 5, as well as S2, S3, S7, and S8 in Table 6. The sample sets of these subjects are not suitable for online MI-BCI classification applications and should be discarded during offline testing. It is worth noting that some subjects exhibit performance degradation from the MTS strategy to the STS strategy, for example, S4 and S7 in Tables 4, 5, and S5, S6, and S9 in Table 6. This indicates that the sample sets of these subjects are not suitable for cross-subject scenarios and often rely on the separability of other subjects' sample sets to achieve outstanding performance under the MTS strategy. Therefore, these subjects should be avoided when training the source domain model for constructing online MI-BCI, and “golden subjects” should be selected instead.

**Table 5:** Cross-subject classification results on BCIIV-2a (F vs. T).

**Subjects**	**STS-R**	**STS-LE**	**STS-E**	**MTS-R**	**MTS-LE**	**MTS-E**
S1	60.85	61.55	60.59	70.14	70.83	65.97
S2	46.88	46.18	55.73	36.11	34.72	45.83
S3	67.19	67.53	67.27	81.94	84.03	81.94
S4	60.76	60.33	61.02	67.36	66.67	69.44
S5	56.77	56.34	60.59	65.28	64.58	64.58
S6	45.83	46.27	49.83	45.83	45.83	48.61
S7	65.36	65.28	68.91	77.08	77.08	78.47
S8	70.92	71.01	81.08	91.67	93.75	95.14
S9	67.10	67.10	69.18	84.72	85.42	81.25
Average	60.18	60.18	**63.80**	68.90	69.21	**70.14**
Standard deviation	8.91	9.02	9.04	18.13	19.08	16.05

The approaches with the highest average accuracy are denoted as bold.

**Table 6:** Cross-subject classification results on BCIIV-2b.

**Subjects**	**STS-R**	**STS-LE**	**STS-E**	**MTS-R**	**MTS-LE**	**MTS-E**
S1	70.53	70.59	70.09	72.75	72.75	73.00
S2	57.66	57.34	57.87	57.75	56.75	59.75
S3	58.13	57.75	57.50	58.75	58.75	60.00
S4	86.63	86.50	87.06	91.00	91.25	92.75
S5	73.06	73.09	71.41	75.50	75.25	71.00
S6	68.94	69.37	69.53	70.50	70.75	71.50
S7	69.31	69.31	68.56	69.00	69.25	67.25
S8	66.50	65.78	66.75	67.50	67.75	67.25
S9	68.53	66.50	67.47	71.75	72.00	72.25
Average	**68.81**	68.47	68.47	70.50	70.50	**70.53**
Standard deviation	8.53	8.68	8.62	9.77	9.98	9.70

The approaches with the highest average accuracy are denoted as bold.

#### 4.4.2 Compared with SOTA methods

To compare the classification results between the proposed method and the SOTA methods, Table 7 illustrates the comparative results based on the MTS strategy, while Table 8 illustrates the comparative results based on the STS strategy. For the FWR-JPDA method, we maintained the same early stopping strategy with *T* = 3 iterations to ensure a fair comparison during the MTS/STS strategy for the three cross-subject MI-EEG classification tasks. As the METL and SB-TA-CSP methods only conducted experiments on the BCIIV-2a (L vs. R) task without open-source codes, we only compared these two methods on the BCIIV-2a (L vs. R) task. The approaches with the highest and the second-highest average accuracy are denoted as bold and underlined, respectively, in the listed results in both tables.

**Table 7:** Comparison of the proposed method and the baseline methods on MTS strategy.

**Methods**	**BCIIV-2a (L vs. R)**	**BCIIV-2a (F vs. T)**	**BCIIV-2b**	**Average**
RA-MDM	72.07 (9.88)	66.28 (17.25)	69.69 (9.70)	69.35
EA-CSP-LDA	73.53 (15.96)	66.90 (16.41)	69.56 (9.43)	70.00
CA-CORAL	72.38 (13.38)	66.82 (16.60)	68.36 (9.42)	69.19
CA-JDA	74.15 (15.77)	67.44 (15.48)	69.19 (9.54)	70.26
MEKT	76.54 (16.72)	70.29 (15.23)	69.42 (9.72)	70.08
METL	76.00 (16.14)	–	–	–
SB-TA-CSP	75.15 (13.52)	–	–	–
FWR-JPDA	77.24 (14.74)	**70.91** (13.70)	70.14 (8.89)	72.76
DS-KTL(proposed)	**78.09** (15.62)	70.14 (16.05)	**70.53** (9.70)	**72.92**

**Table 8:** Comparison of the proposed method and the baseline methods on STS strategy.

**Methods**	**BCIIV-2a (L vs. R)**	**BCIIV-2a (F vs. T)**	**BCIIV-2b**	**Average**
RA-MDM	66.60 (12.60)	59.74 (19.62)	67.28 (9.39)	64.54
EA-CSP-LDA	65.00 (14.06)	59.69 (14.26)	67.49 (9.08)	64.06
CA-CORAL	67.26 (13.34)	59.64 (14.32)	67.50 (9.05)	64.80
CA-JDA	66.59 (15.28)	59.92 (14.28)	67.48 (9.14)	64.66
MEKT	68.73 (15.73)	60.11 (7.85)	66.17 (8.10)	65.00
METL	69.06 (15.50)	–	–	–
SB-TA-CSP	68.76 (9.61)	–	–	–
FWR-JPDA	67.48 (15.23)	65.67 (12.68)	**68.59** (9.34)	67.25
DS-KTL(proposed)	**71.61** (14.23)	**63.80** (9.04)	68.47 (8.62)	**67.96**

Based on the results presented in Table 7, it can be deduced that our DS-KTL method achieved the best classification performance for both the BCIIV-2a (L vs. R) and BCIIV-2b tasks. However, it slightly underperfored compared to the FWR-JPDA method on the BCIIV-2a (F vs. T) task. Overall, our method achieved the highest average classification performance across the three tasks under the MTS strategy. Similarly, as observed from Table 8, our method demonstrated the best classification performance for both the BCIIV-2a (L vs. R) and BCIIV-2a (F vs. T) tasks, while slightly underperforming compared to the FWR-JPDA method for the BCIIV-2b task. Overall, our method also achieved the highest average classification performance across the three datasets under the STS strategy.

As a common practice (Zhang and Wu, 2020b; Mishuhina and Jiang, 2021), dividing *n* classes into n*(*n* − 1)/2 binary classification tasks is a basic strategy. In our DS-TKL method, since the pseudo-label selection classifier is only applicable to binary classification problems, to compare the FOUR-class classification results, we divided the four MI tasks (L, R, F, and T) in the BCIIV-2a dataset into six binary classification tasks: Task 1 (L vs. R), Task 2 (L vs. F), Task 3 (L vs. T), Task 4 (R vs. F), Task 5 (R vs. T), and Task 6 (F vs. T). Due to space limitations, we only present the average classification results for the six binary tasks in Tables 9, 10, other results and ablation studies are given for two representative tasks of Task 1 and Task 6. It should be noted that the “Average” and “Standard Deviation” in Tables 9, 10 are derived from the average values and standard deviations of the six tasks in each column.

**Table 9:** Six binary classification tasks on BCIIV-2a compared within the STS strategy.

**Tasks**	**MEKT**	**FWR-JPDA**	**DS-TKL (proposed)**
Task 1	68.73 (15.73)	67.48 (15.23)	71.61 (14.23)
Task 2	64.10 (8.93)	65.05 (16.92)	68.34 (12.28)
Task 3	70.88 (11.62)	73.18 (16.56)	76.71 (13.99)
Task 4	63.97 (8.42)	60.88 (19.33)	65.84 (12.55)
Task 5	69.12 (9.66)	69.48 (18.63)	73.22 (13.17)
Task 6	60.11 (7.85)	65.67 (12.68)	63.80 (9.04)
Average	66.15	66.96	**69.92**
Standard deviation	4.08	4.19	4.83

The approaches with the highest average accuracy are denoted as bold.

**Table 10:** Six binary classification tasks on BCIIV-2a compared within the MTS strategy.

**Tasks**	**MEKT**	**FWR-JPDA**	**DS-TKL (proposed)**
Task 1	76.00 (17.61)	77.01 (12.91)	78.09 (15.62)
Task 2	73.07 (15.43)	75.69 (13.35)	74.23 (16.47)
Task 3	81.10 (16.99)	80.56 (15.59)	80.86 (17.32)
Task 4	72.99 (15.50)	74.07 (14.99)	73.23 (17.39)
Task 5	79.94 (15.58)	78.47 (17.22)	79.01 (18.71)
Task 6	69.98 (7.73)	70.06 (15.76)	70.53 (9.70)
Average	75.51	75.98	**75.99**
Standard deviation	4.34	3.66	3.94

The approaches with the highest average accuracy are denoted as bold.

According to the experimental settings, the MTS strategy produces a total of nine cross-subject tasks, and we present the average classification results for each subject as the target domain among Task 1 to Task 6 in Table 10. The MTS strategy generates 9*8 = 72 cross-subject classification tasks in total. For each subject acting as the target domain, the remaining eight subjects serve as the source domains. The classification accuracies obtained from these eight source domains are averaged to calculate the average classification accuracy of the target domain under the STS strategy. Table 9 provides the average classification results for each subject as the target domain among Task 1 to Task 6. From the results in the tables, it can be observed that our DS-KTL method consistently outperforms the two compared SOTA methods under both the MTS and STS strategies. The average classification accuracy under the STS strategy significantly improves across the six tasks, while the improvement in average classification accuracy under the MTS strategy is less pronounced. Moreover, the standard deviation of the six classification tasks is comparable to the SOTA methods, indicating that the algorithm exhibits similar generalization performance across different tasks and possesses good robustness.

#### 4.4.3 Feature visualization

**Figure 3:**
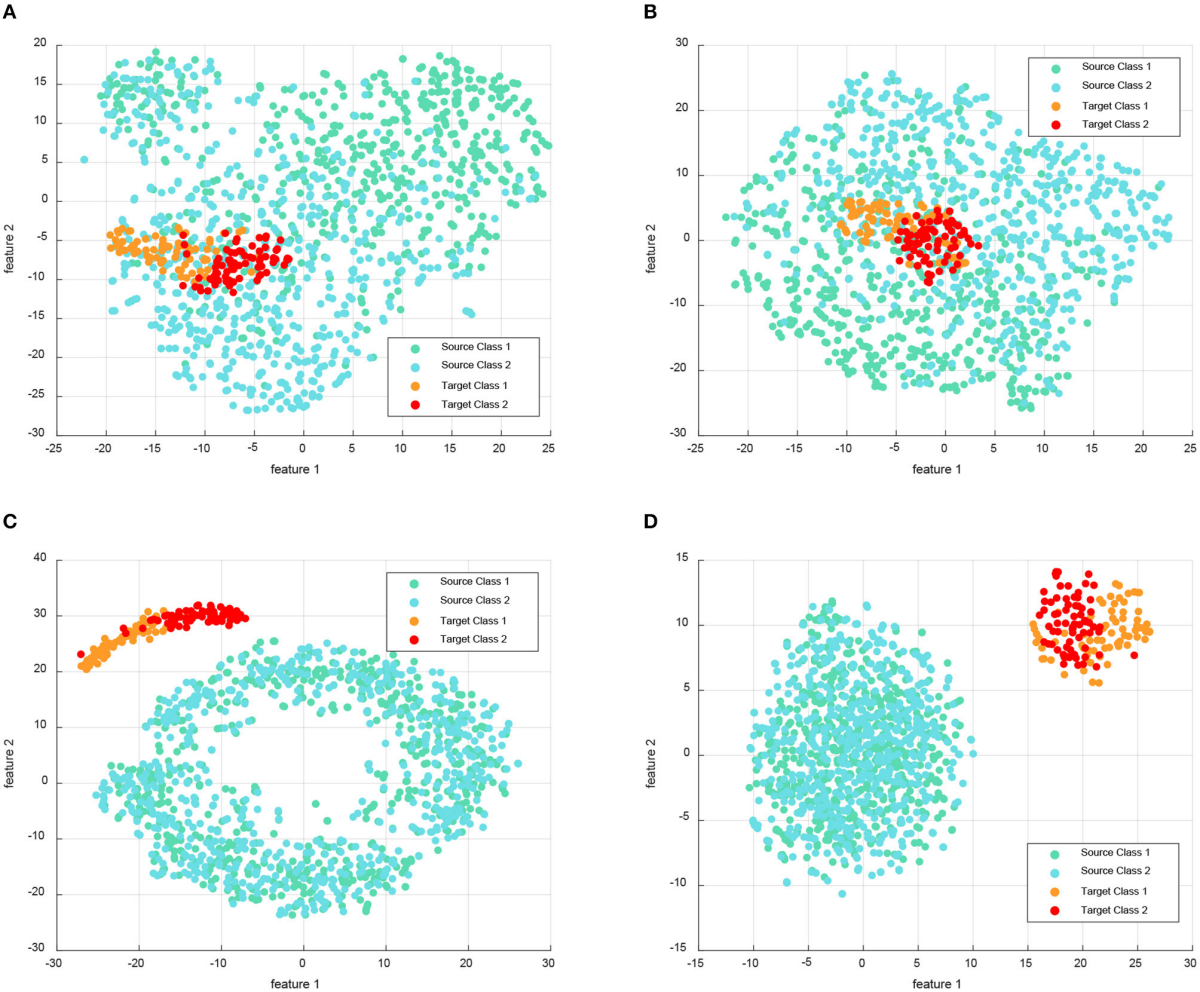
Feature visualization compared with the SOTA for the Subject 1 as the target domain when using MTS strategy on BCIIV-2a dataset. **(A)** CA-CORAL. **(B)** MEKT. **(C)** FWR-JPDA. **(D)** DS-KTL (proposed).

In addition to quantitative comparisons of accuracy, we also provide qualitative comparisons of feature visualization between different methods. For visualization, we select Subject 1 as the target domain when using the MTS strategy on the BCIIV-2a (L vs. R) task. During the feature adaptation process, we visualize the features of the two MI classes (left hand MI task represented by class 1 and right hand MI task represented by class 2) in both the source and target domains. To facilitate visualization, we reduce the feature dimensions to 2 using the t-SNE tool (Van der Maaten and Hinton, 2008). Figure 3 presents the feature visualization comparison between our DS-KTL method and three SOTA methods. In the figure, class 1 of the source domain is represented by green dots, class 2 of the source domain is represented by blue dots, class 1 of the target domain is represented by orange dots, and class 2 of the target domain is represented by red dots.

Based on the observations from Figure 3, we can deduce that centroid alignment aligns the covariance of samples from different subjects, whitening them into similar distributions and reducing the distribution discrepancy across subjects. In contrast, classical methods such as CA-CORAL and MEKT do not differentiate between the source and target domain samples. However, after feature adaptation, the model trained on the source domain can effectively discriminate different classes in the target domain, providing accurate pseudo-labels to ensure classification accuracy. On the other hand, the FWR-JPDA method achieves sample separation between the source and target domains through dual regularizations while simultaneously maintaining the similarity of sample distributions. Consequently, it can successfully apply the model trained from the source domain to the target domain. Also, our DS-KTL method selects discriminative features and samples with correct pseudo labels for adaptation, achieving samples separation between the source and target domains, too.

Formally, cross-domain adaptation requires training the classifier on the source domain to ensure its high generalization performance on the target domain. The visualization shown in Figure 3 presents the t-SNE reduction of features to two dimensions, which illustrates the distribution of the source and target domains. The classifier trained on the original higher-dimensional features exhibits separability among different classes, which may not be directly discernible in the reduced feature space. In cross-subject MI-EEG classification, for a classifier trained on the source domain to achieve high accuracy on the target domain, it is necessary to ensure similarity in the distribution of the two domains' reduced feature sets. In Figure 3, a higher similarity in the distribution of the source and target domains is observed for the METK method and our DS-KTL method, compared to the CA-CORAL and FWR-JPDA methods. Furthermore, as shown in the sample distribution in Figure 3D, our DS-KTL method, which incorporates feature selection, shows a distribution of selected features that is closer to both domains compared to the METK method. Although it achieves a lower distance in differentiating the two domains, it effectively enables the classifier trained on the source domain to have good generalization performance on the target domain.

**Figure 4:**
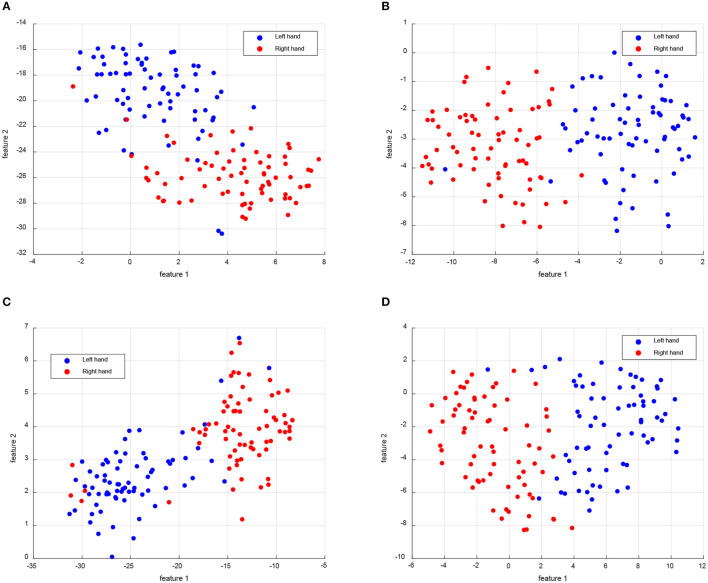
Feature visualization compared with the SOTA for the left hand and right hand classes of Subject 8 as the target domain when using MTS strategy on BCIIV-2a dataset. **(A)** CA-CORAL. **(B)** MEKT. **(C)** FWR-JPDA. **(D)** DS-KTL (proposed).

However, Figure 3 only presents the distribution of the source and target domains, without clear discrimination of samples for each class. In order to demonstrate the ability to discriminate different classes in a new subject, we selected Subject 8 as the target domain in the MTS strategy of Dataset 2a (L vs. R) dataset. To this end, we visualized the distribution of left hand and right hand samples in a two-dimensional feature space using the t-SNE tool. The resulting samples distribution is depicted in Figure 4. From the results shown in Figure 4, it can be observed that all four algorithms demonstrate good discrimination between the left hand and right hand classes. Specifically, our proposed DS-KTL method exhibits the best discrimination ability, effectively distinguishing between the two classes with a relatively uniform feature distribution. The sample distribution of CA-CORAL and FWR-JPDA methods appear to be uneven. The CA-CORAL method exhibits closely located features for two classes, leading to classification difficulties, while the FWR-JPDA method encounters issues with mixed class distributions, with some right hand samples appearing in the left hand distribution and vice versa. Although MEKT also supports favorable feature distribution, it contains two left hand samples in the right hand distribution, which reduces the classification accuracy. The feature selection and pseudo-labels selection employed in our proposed DS-KTL method effectively identify outlier points from other classes in the sample distribution, thereby enhancing the performance of cross-subject MI-EEG classification.

#### 4.4.4 Efficiency

Efficiency is another crucial metric for evaluating cross-subject MI-EEG classification methods, as it determines their suitability for constructing online MI-BCI systems. To facilitate efficiency comparison, Tables 11, 12 present the time complexity comparison for six cross-subject MI-EEG classification tasks under the MTS and STS strategies, respectively. For each dataset, we report the average runtime for nine subjects, representing the average time consumption for the nine tasks under the MTS strategy and the 72 tasks under the STS strategy. Based on the results in Tables 11, 12, it can be ascertained that classical sample alignment and feature adaptation methods exhibit lower time complexity. These methods do not involve the iterative process of obtaining pseudo-labels and achieve only limited classification performance by aligning the marginal distribution or subspace obtained from the target domain.

**Table 11:** Time complexity comparison for the MTS strategy (unit:s).

**Methods**	**BCIIV-2a (L vs. R)**	**BCIIV-2a (F vs. T)**	**BCIIV-2b**	**Average**
RA-MDM	0.99	0.98	0.57	0.85
EA-CSP-LDA	0.56	0.57	0.58	0.57
CA-CORAL	0.56	0.56	0.50	0.54
CA-JDA	7.27	7.04	71.80	28.70
MEKT	0.49	0.44	0.79	0.57
FWR-JPDA	0.96	0.88	6.30	2.71
DS-KTL (proposed)	1.47	1.57	19.50	7.51

**Table 12:** Time complexity comparison for the STS strategy (unit:s).

**Methods**	**BCIIV-2a (L vs. R)**	**BCIIV-2a (F vs. T)**	**BCIIV-2b**	**Average**
RA-MDM	0.192	0.19	0.12	0.17
EA-CSP-LDA	0.088	0.086	0.081	0.085
CA-CORAL	0.086	0.089	0.084	0.086
CA-JDA	0.40	0.44	1.89	0.91
MEKT	0.34	0.34	0.24	0.31
FWR-JPDA	0.089	0.084	0.18	0.12
DS-KTL (proposed)	0.34	0.30	0.29	0.31

The JDA method was the first to employ pseudo-labels from the target domain to obtain conditional distribution for feature adaptation, resulting in higher time complexity. When comparing the BCIIV-2a and BCIIV-2b datasets, it becomes evident that the larger number of samples leads to increased time consumption during iterations. In contrast, the MEKT/FWR-JPDA/DS-KTL methods rely on the JPDA method, which exhibit lower time complexity and require fewer iterations while ensuring alignment of conditional distribution to achieve decent performance. Overall, whether under the MTS or STS strategy, our DS-KTL method demonstrates a similar order of time complexity as SOTA methods. The time consumption of DS-KTL primarily arises from manifold embedded spatial feature selection. Under the MTS strategy, feature selection takes place in the source domain, and considering the BCIIV-2b dataset with 400*8 = 3, 200 samples, it requiring more time for selection. In contrast, for the smaller BCIIV-2a dataset and STS strategy, the time complexity of feature selection is not high. Our DS-KTL method showcases comparative efficiency compared to SOTA methods, as the selected pseudo-labels with high probability help decrease the time complexity during iterative feature adaptation.

### 4.5 Ablation study

To validate the feasibility and effectiveness of our DS-KTL method, we have conducted three ablation studies to assess its characteristics.

#### 4.5.1 Performance of different selections

**Figure 5:**
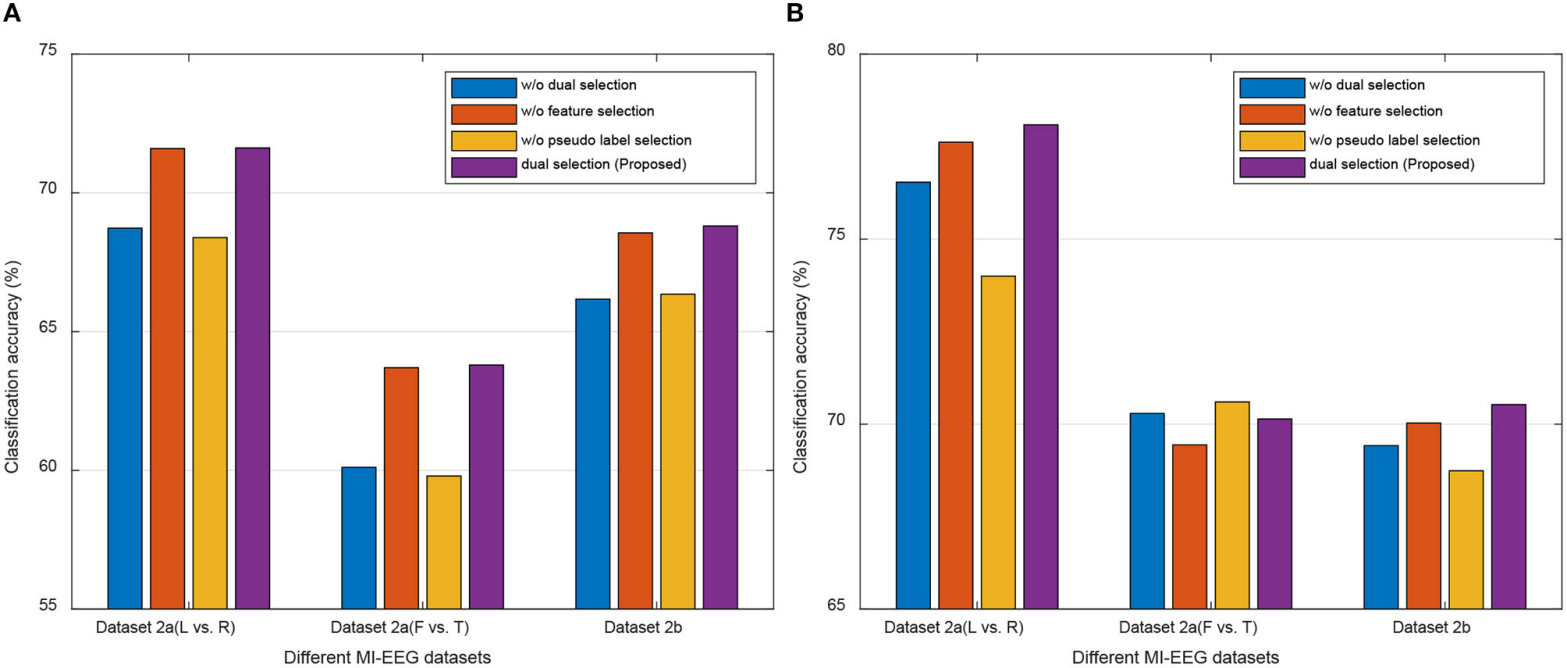
Ablation studies for the performance comparison of different selections. **(A)** MTS strategy. **(B)** STS strategy.

Our DS-KTL method incorporates dual selections during feature adaptation. To validate the impact of these dual selections on the performance of cross-subject MI-EEG classification, we conducted ablation studies by removing the selections on the three MI-EEG classification tasks under the MTS/STS strategy. The ablation studies included the following scenarios: (1) with/without (w/o) dual selections; (2) w/o feature selection; (3) w/o pseudo label selection; (4) with dual selections. Figure 5 illustrates the results of ablation studies, allowing for performance comparison among the different selection scenarios.

Based on the results depicted in Figure 5, we can finalized that, except for the Dataset 2a (F vs. T) task under the MTS strategy, our proposed DS-KTL outperforms the three “without selection” settings in the other five tasks. When comparing feature selection and pseudo-label selection, the former demonstrates superior performance improvement, particularly for Dataset 2a, which possesses higher-dimensional spatial features. Manifold embedded feature selection allows for the extraction of more discriminative feature combinations, resulting in higher accuracy in cross-subject MI-EEG classification. Furthermore, using pseudo-labels selection alone shows minimal impact on performance improvement and may even hinder classification performance, especially in the Dataset 2a (L vs. R) and Dataset 2b tasks under the MTS strategy when compared to the case without selection. However, by integrating dual selections, our proposed DS-KTL method exhibits substantial enhancement in classification performance. Therefore, we can conclude a verdict that the dual selection processes complement each other and are indispensable components of our method.

In the MTS strategy, only the “Dataset 2a (F vs. T)” classification task shows “w/o pseudo label selection” exceeding our proposed method. This is because the “feet vs. tongues” class in MI itself is inherently difficult to distinguish, resulting in lower recognition accuracy. Therefore, in the initially domain adaptation phase, the target subject generates more wrongly labeled pseudo labels, which decreases the accuracy of domain adaptation. In summary, the pseudo label selection strategy used in this study is not suitable for difficult-to-classify classes, as the erroneous samples generated by the target subject will hinder the overall accuracy.

In fact, for the STS strategy, the feature selection and pseudo-label selection shows differences across different subjects. To validate the variations of such two selections under the STS strategy, we have conducted the experimental results of the ablation study for each subject as the target domain in Table 13. The results will instruct us in identifying the specific subjects' outcomes regarding feature selection and pseudo-label selection. In Table 13, “w/o PLS” refers to “without pseudo label selection,” “w/o FS” refers to “without feature selection,” and “DS” stands for the proposed “dual selection.” For each subject considered as the target domain, the table provides the average classification accuracy when the remaining 8 subjects are used as the source domain.

**Table 13:** Ablation studies compared of each subject as the target domain under STS strategy.

**Target**	**BCIIV**	**2a**	**(L vs. R)**	**BCIIV**	**2a**	**(F vs. T)**	**BCIIV**	**2b**	
**Subject**	**w/o PLS**	**w/o FS**	**DS**	**w/o PLS**	**w/o FS**	**DS**	**w/o PLS**	**w/o FS**	**DS**
S1	75.87	79.77	79.60	58.94	60.42	61.20	67.91	70.94	69.78
S2	51.48	54.69	54.08	47.05	55.03	55.30	57.03	57.38	57.88
S3	83.16	85.42	85.33	67.01	68.06	69.62	57.28	57.34	57.63
S4	66.23	67.71	67.71	58.68	59.90	59.20	82.56	86.03	87.38
S5	53.65	57.03	57.64	56.68	61.02	60.68	69.14	71.75	72.16
S6	64.24	63.19	63.11	51.39	50.09	48.70	68.31	69.16	69.72
S7	61.72	66.84	66.06	60.85	69.27	68.84	62.41	68.94	68.75
S8	86.72	90.19	90.19	73.78	77.78	78.13	65.09	66.78	66.81
S9	72.48	78.21	77.95	63.02	69.36	70.83	68.94	66.78	67.31

From the results in Table 13, we observed that pseudo-label selection contributes more to the improvement in recognition accuracy compared to feature selection. When both are combined as dual selection, some specific subjects show improvements in accuracy, for example, S5 in Dataset 2a (L vs. R), S1, S3, S8, and S9 in Dataset 2a (F vs. T), and S4, S5, and S9 in Dataset 2b. However, it is important to note that, dual selection, which combines feature selection with pseudo-label selection, can suppress the accuracy of certain subjects, for instance, S2, S7, and S9 in Dataset 2a (L vs. R), S4, S5, and S6 in Dataset 2a (F vs. T), and S1 and S7 in Dataset 2b. Overall, from the average values in Figure 5B, we can see that there is not much difference in average accuracy between “w/o FS” and “DS” in the STS strategy, indicating performance differences on specific subjects.

For the results presented in Table 13 under the STS strategy, we conducted *t*-test statistical hypothesis tests to examine whether each subject, when serving as the target domain, exhibited a significant improvement in MI-EEG classification accuracy. Using our proposed DS-KTL (DS) as the baseline, we performed statistical hypothesis tests for “w/o PLS vs. DS” and “w/o FS vs. DS”. When the test results in *p* < 0.1, *p* < 0.05, and *p* < 0.01, the corresponding subjects are marked with ^*^, ^**^, and ^***^, respectively. Table 14 provides the *t*-test statistical hypothesis results with corresponding *p*-values for each subject under the STS strategy.

**Table 14:** *T*-test statistical hypothesis results with corresponding *p*-values for each subject under the STS strategy.

**BCIIV-2a**	**(L vs. R)**	**BCIIV-2a**	**(F vs. T)**	**BCIIV**	**2b**
**w/o PLS vs. DS**	*p* **-value**	**w/o PLS vs. DS**	*p* **-value**	**w/o PLS vs. DS**	*p* **-value**
*S*1^*^	0.097	*S*2^*^	0.084	*S*1^*^	0.074
*S*2^*^	0.088	*S*5^***^	0.0013	*S*4^**^	0.022
*S*5^*^	0.099	*S*7^*^	0.073	*S*5^***^	0.001
*S*7^*^	0.070	*S*9^**^	0.033	*S*7^***^	0.0098
**w/o FS vs. DS**	*p* **-value**	**w/o FS vs. DS**	*p* **-value**	**w/o FS vs. DS**	*p* **-value**
–	–	*S*6^**^	0.030	*S*1^**^	0.040
–	–	*S*9^*^	0.095	–	–

According to Table 14, it can be observed that, compared to not using pseudo-labels selection (w/o PLS), our proposed DS-KTL method exhibits significant performance improvements on subjects S1, S2, S5, and S7 in Dataset 2a (L vs. R), as well as on subjects S2, S5, S7, and S9 in Dataset 2a (F vs. T), and subjects S1, S4, S5, and S7 in Dataset 2b. Regarding not using feature selection (w/o FS), significant performance improvements are only observed on subjects S2, S5 in Dataset 2a (F vs. T), and subject S1 in Dataset 2b. Overall, our proposed DS-KTL method demonstrates significant performance improvements compared to w/o PLS on 12 out of 27 subjects in Dataset 2a and 2b, while compared to w/o FS, it shows significant performance improvements on only three out of 27 subjects. Thus, the effect of pseudo-labels selection is more pronounced under the STS strategy, whereas the improvement from feature selection is limited.

#### 4.5.2 Number of selected features

**Figure 6:**
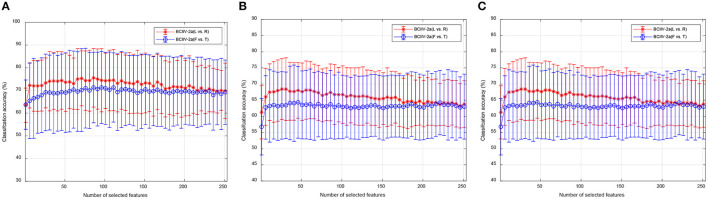
Ablation studies for the performance comparison for different numbers of feature selection. **(A)** BCIIV-2a under MTS strategy. **(B)** BCIIV-2a under STS strategy. **(C)** BCIIV-2b.

The aforementioned ablation study has confirmed that manifold embedded feature selection is capable of selecting spatial features that are more advantageous for cross-subject MI-EEG classification. To further validate the optimal range of feature dimension selection, we assessed the impact of different numbers of feature selections on classification performance. We performed experiments on selected features number of *q* = 1, 2, .., 253 and *q* = 1, 2, , 6 for BCIIV-2a and BCIIV-2b datasets, respectively. Figure 6 illustrates the influence of different numbers of feature selection on the final classification accuracy under the MTS/STS strategy. The average accuracy and standard deviation of cross-subject MI-EEG classification were provided for each feature number. Since the BCIIV-2a dataset had more results available, and there was not a significant difference when increased or decreased less number of features, we presented the results every five features, providing ~50 showcase results. It is worth noting that due to the variation in the selected feature numbers, the dimension for feature adaptation in KTL was set as *z* = *q*, following the experimental setup.

From the results in Figure 6, it can be determined that both the MTS and STS strategies are insufficient to achieve high recognition accuracy when the selected number of features is small, especially for *q* < 5. As the number of selected features increased, the classification accuracy gradually stabilized with little variation. The standard deviation of the classification performance remained around 10 when selecting different numbers of features for different datasets. Overall, selecting a smaller number of features already provides high classification accuracy for MTS and STS tasks on the BCIIV-2a dataset (*q* < 50). Usually, redundant features may cause negative transfer during feature adaptation, and selecting discriminative features with appropriate number could reduce time complexity. For the BCIIV-2b dataset, excellent performance could already be achieved with *q* = 2, while both MTS and STS strategies achieved optimal performance at *q* = 5, following a similar pattern to the BCIIV-2a dataset.

**Figure 7:**
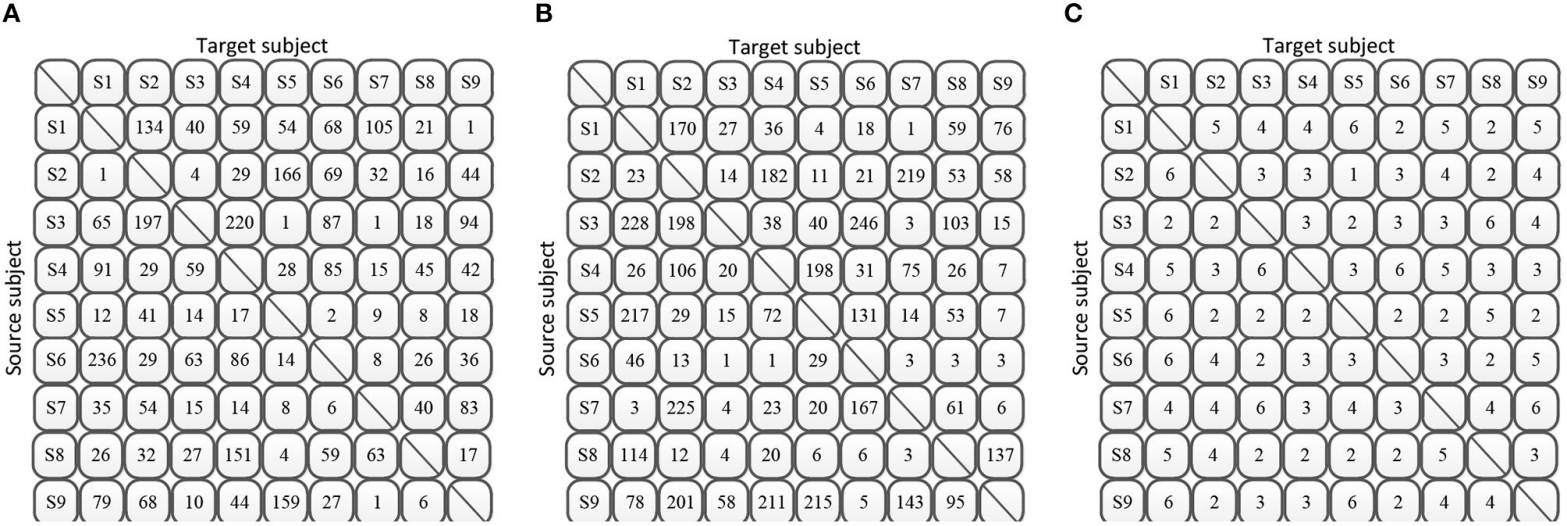
Optimal number of selected features for each source subject and target subject under STS strategy. **(A)** BCIIV-2a (L vs. R). **(B)** BCIIV-2a (F vs. T). **(C)** BCIIV-2b.

Moreover, in the MTS approach, we considered *S* − 1 subjects as a single entity, thus allowing only a fixed number *q* of selected features. However, in the STS approach, we should determine the optimal feature dimensions selection for each source subject. Through experiments, we presented the experimental results of the optimal feature selection for each source subject on each target subject in Figure 7. The number in the figures is the selected number of features when achieveing the best classification accuracy with respect to the specific source subject and target subject.

From the results in Figure 7, we can observe that under the STS strategy, different source subjects have different optimal selected features of number *q* when serving as the source domain. In cases where there is high feature discriminability, the optimal accuracy corresponds to a lower number of selected features. On the other hand, for more challenging classification scenarios, a higher number of feature dimensions is required to achieve the optimal accuracy. Figures 7A,B show that the majority of source subjects in Dataset 2a require fewer than 100 feature dimensions to achieve the optimal accuracy. The results depicted in Figure 7C also indicate that the majority of source subjects select fewer than 6 dimensions in Dataset 2b to achieve the optimal accuracy. This further demonstrates the feasibility of feature selection, as selecting a dimensionality lower than the maximum number of features yields the best classification accuracy.

#### 4.5.3 Parameters sensitivity

According to the proposed DS-KTL method, three sets of parameters have been introduced: α, β, γ in the feature selection, λ, *z, T* in the feature adaptation, and η, μ, σ in the regularizations during knowledge transfer learning. To measure the parameter sensitivity, we conducted ablation studies for each set of parameters. In each experiment, we maintained the default settings of the other remaining parameters introduced in Section 4.3 and Table 3, and tuned the specific parameter under consideration for the cross-subject MI-EEG classification of the six tasks.

**Figure 8:**
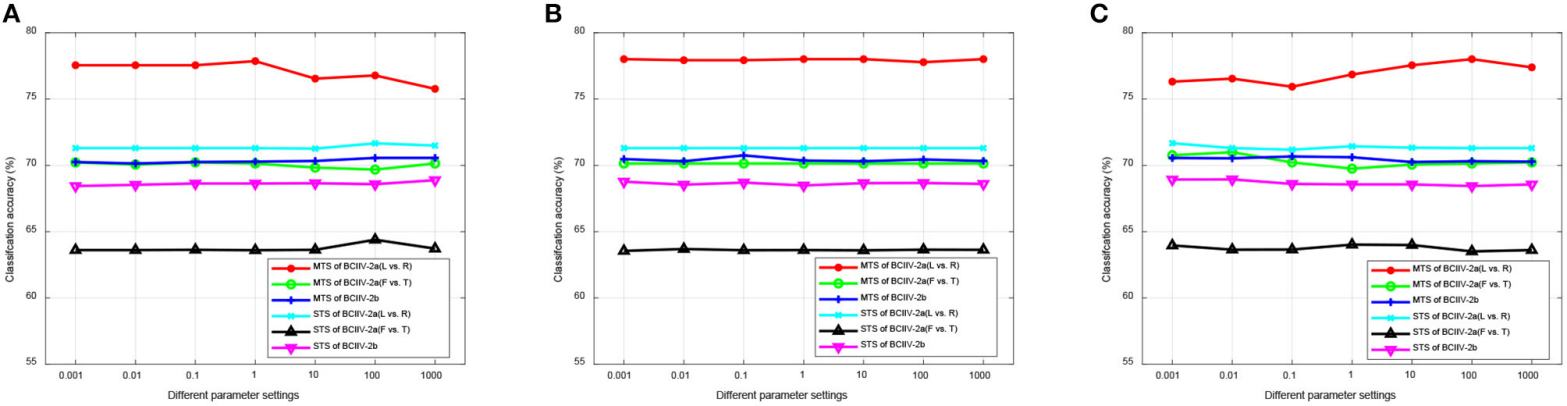
Parameters sensitivity of manifold embedded features selection. **(A)** α. **(B)** β. **(C)** γ.

In the first experiment, we tuned each parameter of α, β, γ within a range of {0.001, 0.01, 0.1, 1, 10, 100, 1, 000} while keeping the other two parameters at their default settings. Figure 8 illustrates the results of parameters sensitivity experiment for manifold embedded features selection. From Figure 8, it can be observed that the manifold embedded spatial feature selection method is not highly sensitive to parameter settings. Different parameter ranges have minimal impact on the selection of discriminative features. This demonstrates good robustness across different datasets and MTS/STS strategies, making it suitable for constructing MI-BCI applications in practical scenarios.

**Figure 9:**
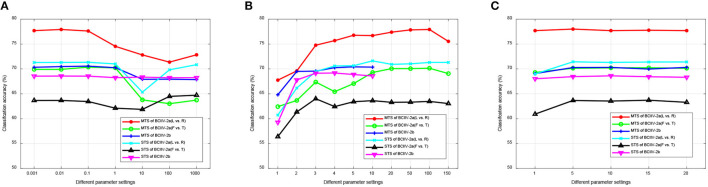
Parameters sensitivity of feature adaptation. **(A)** λ. **(B)** *z*. **(C)** *T*.

For the second experiment, we focused on the parameterse used in the JPDA method, which is employed in our DS-KTL method for feature adaptation. These parameters include λ to counterbalance transferability and discriminability, *z* to determine the subspace for alignment, and *T* for iteratively predicting pseudo-labels. In this experiment, we tuned λ within a range of {0.001, 0.01, 0.1, 1, 10, 100, 1, 000}. Besides, *z* was tuned within a range of {1, 2, 3, 4, 5, 10, 20, 50, 100, 150}, and *T* was tuned within a range of {1, 5, 10, 15, 20}. Figure 9 illustrates the the results of parameters sensitivity experiment for feature adaptation.

Based on the observations from Figure 9, it can be inferred that during the iterative process of feature adaptation, transferability takes higher precedence over discriminability. This prioritization leads to higher average classification accuracy when λ < 1. As λ continues to increase, it leads to a decline in classification performance. Furthermore, a higher dimension of subspace is preferred, as an increase in *z* improves the average classification performance. However, it is important to note that higher dimensions require more time during the iteration process. While they ensure better performance, efficiency decreases accordingly. Based on the experimental results across the six MI-EEG classification tasks, selecting a dimension of *z* = 10 strikes a good balance between performance and efficiency. Additionally, the experimental results regarding the number of iterations indicate that higher iteration counts have minimal impact on the final results. Due to the adoption of the early stopping strategy in our DS-KTL method, higher iteration counts do not necessarily result in improved performance. Instead, they lead to higher time complexity.

**Figure 10:**
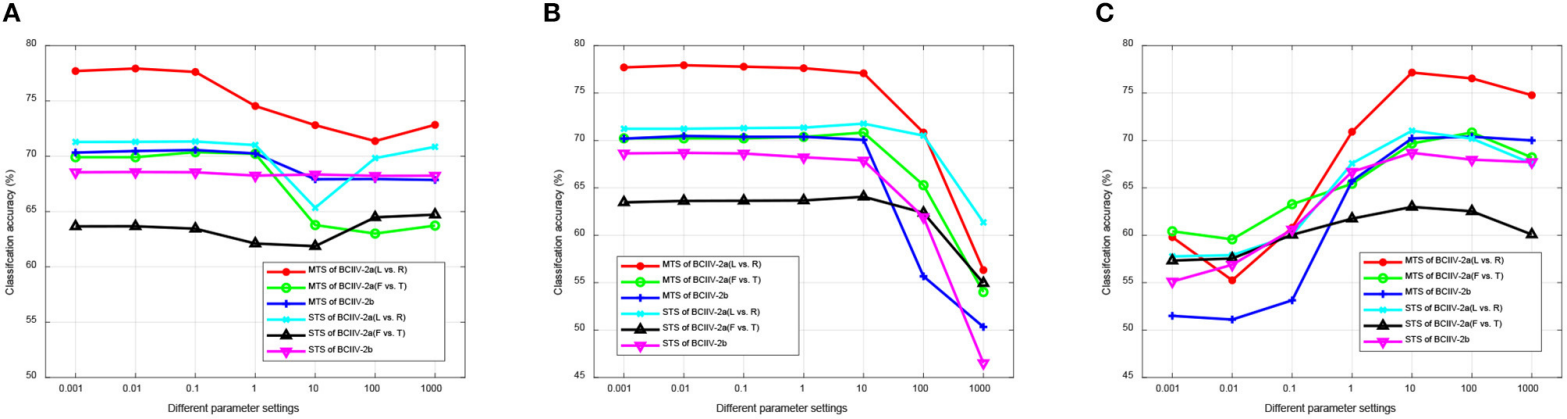
Parameters sensitivity of regularizations during knowledge transfer learning. **(A)** η. **(B)** μ. **(C)** σ.

For the third experiment, we tuned each parameter η, μ, σ wihtin a range of {0.001, 0.01, 0.1, 1, 10, 100, 1, 000} while keeping the other two parameters at their default settings. Figure 10 illustrates the results of parameters sensitivity experiment for the regularizations during knowledge transfer learning. From Figure 10, it can be concluded that setting smaller values for the regularizations related to the discriminability of the source domain and the locality preservation of the target domain ensures higher average classification accuracy. On the other hand, assigning larger parameter values leads to a rapid decline in classification performance. However, for the regularization of the transformation vector, the average classification accuracy initially increases and then decreases as the parameters increase. The highest classification accuracy is achieved at σ = 10. This pattern mainly occurs because the values of the transformation vector significantly differ in magnitude from the other regularizations, necessitating higher parameter values for appropriate adjustment.

## 5 Discussion

### 5.1 Superiority

In cross-subject MI-EEG classification, effectively representing time-varying and spatially coupled sample distributions is crucial as it plays a decisive role in reducing the data distribution discrepancy between the source and target domains (Liu et al., 2023; She et al., 2023). Currently, CSP and RTS have been applied for feature representation (He and Wu, 2019; Cai et al., 2022), while feature adaptation is employed to minimize the distribution discrepancy. Recently, the introduction of conditional distribution in feature adaptation is an innovative step (Long et al., 2013; Zhang and Wu, 2020b), but the lack of knowledge about the labels in the target domain limits us to using pseudo-labels, leading to error accumulation during the iterative process (Wang and Breckon, 2020; Yue et al., 2021). Additionally, the redundant spatial feature representation is not well-suited for minimizing the distribution discrepancy between the source and target domains (Ren et al., 2022). In this study, the proposed dual selection addresses the aforementioned issues by utilizing supervised MEFS for selecting appropriate features and assuming their superiority in the target domain. Experimental results demonstrate that spatial feature selection indeed improves the performance of domain adaptation, except for the Dataset 2a (F vs. T) task (see Figure 5).

For feature selection, commonly used methods include mutual information and computational intelligence methods (Kumar et al., 2017; Kirar and Agrawal, 2020; Luo, 2023), which may suffer from suboptimal feature selection or uncertainty during iterations. In our feature selection method, MEFS, based on the experimental results, we could reduce the number of features to about half their original number. This enhances discriminability during the feature adaptation process and improves efficiency (see Figure 6). Pseudo-label selection for the target domain has been mainly applied in cross-domain tasks (Li et al., 2023; Wang and Zhang, 2023). In our approach, we employ this strategy and combine it with spatial feature selection, which leads to improved cross-subject MI-EEG classification performance (see Figure 5). Furthermore, the experiments also demonstrate that label distribution discrepancy caused by cross-subject variations can lead to performance degradation when relying solely on pseudo-label selection. Label alignment (He and Wu, 2020) may be one of the potential future directions to address this limitation in our method.

Centroid alignment, as a classical cross-subject preprocessing method (Zhang and Wu, 2020b), is particularly crucial for computing the covariance centroid. Previous research has shown that different subject sample distributions correspond to different metric approaches (Li et al., 2021). Our experimental results confirm this finding, as the BCIIV-2a dataset is better suited for Euclidean metric in our DS-KTL method, both for MTS and STS strategies (see Tables 4, 5). The BCIIV-2b dataset is known to be more challenging (Mishuhina and Jiang, 2021), and our DS-KTL method exhibits little difference in alignment metrics for this dataset, both for MTS and STS strategies (see Table 6).

Moreover, the time complexity is an important metric for evaluating the applicability of classification methods in online MI-BCI systems. Studies have indicated (Arpaia et al., 2022; Tao et al., 2023) that achieving stable online MI-BCI control performance with millisecond-level response is crucial for generating control commands. Our DS-KTL method demonstrates competitive time complexity (see Tables 11, 12). For instance, on the target domains of dataset BCIIV-2a within 144 MI-EEG samples, the cross-subject MI-EEG classification using MTS or STS strategies can be completed in ~1.47 and 0.34 s, respectively. This achievement is close to reaching the performance level required for online MI-BCI and contributes to the development of rehabilitation systems. Feature selection is the main time-consuming task, as confirmed by previous research (Luo, 2023), especially when dealing with a large number of source domain samples that influence the optimal feature number. Reducing the number of source domain samples is a focal point of our future research. Based on our experimental results, we can identify a robust “golden subject” (Sun et al., 2022) that requires fewer source domain samples while enhancing discriminability. This approach can significantly reduce the time complexity of MEFS (see Tables 4–6).

Furthermore, compared to conventional binary classification SOTA methods without feature selection or pseudo-labels selection, our DS-KTL method achieves improved cross-subject classification performance on Dataset 2a (L vs. R), Dataset 2a (F vs. T), and Dataset 2b for both the MTS and STS strategies (see Tables 7, 8). Among them, for the STS strategy, the *t*-test results revealed significant classification improvements for our proposed dual-selection strategy compared to a single selection strategy in four subjects of BCIIV-2a (L vs. R) and BCIIV-2b, as well as in six subjects of BCIIV-2a (F vs. T; see Table 14). Additionally, for the binary classification tasks among Task 1 to Task 6, which are derived from the four classes in Dataset 2a, our DS-KTL method demonstrates significantly higher classification performance than the METK (Zhang and Wu, 2020b) and FWR-JPDA (Zhang and Wu, 2020b) methods under the STS strategy (see Tables 9, 10). However, under the MTS strategy, the classification performance of DS-KTL is comparable to that of METK and FWR-JPDA. According to the experimental settings, the “Task” of STS strategy treats each subject as target domain and another subject as source domain, therefore results in 8*9 = 72 sub-tasks from nine subjects. Meanwhile, the “Task” of MTS strategy treats the remain subjects as source domain and results in eight sub-tasks. So we think the main reason is that 72 sub-tasks under the STS strategy will benefited from the dual selection than the nine sub-tasks under the MTS strategy, therefore achieved a higher average classification performance. This further emphasizes the importance of feature selection and pseudo label selection when using the STS strategy in resource-limited online BCI control scenarios.

Regarding the sample distribution, we selected representative source and target domains and provided experimental results on the sample distribution, which subjectively support the objective classification accuracy results (see Figure 3). Parameter sensitivity is another important metric for evaluating cross-subject MI-EEG classification algorithms (Arpaia et al., 2022), as it determines whether they can be effectively applied in online MI-BCI applications. In our DS-KTL method, we have a total of nine parameters, including MEFS, feature adaptation, and regularizations. Based on our experimental results, all parameter settings within appropriate ranges exhibit insensitivity (see Figures 8–10). Our experiments conducted parameter sensitivity tests on three tasks across two datasets, demonstrating the robustness of the cross-subject sample set.

### 5.2 Limitation

Although our DS-KTL method achieves high performance in cross-subject classification on the selected representative MI-EEG datasets, there are still areas where further improvements can be made. Firstly, spatial domain features struggle to capture the comprehensive characteristics of nonlinear and non-stationary MI-EEG signals. Recent researches have started to divide MI-EEG signals into time-frequency segments (Mishuhina and Jiang, 2021; Luo, 2023) and extract CSP or RTS features from different time segments and frequency bands. The resulting feature combinations encompass temporal-spatial-spectral characteristics, thus overcoming the limitations of classical CSP or RTS features.

Secondly, if more comprehensive temporal-spatial-spectral feature representations are adopted, feature adaptation will also face the limitations of high-dimensional features, making effective feature selection particularly important. Experimental findings reveal that our proposed supervised MEFS method experiences an increase in time complexity as the number of source domain samples increases, while it cannot guarantee optimal feature selection on the target domain, thus exhibiting limitations in both performance and efficiency. Recently, researchers have proposed efficient and unsupervised multi-label feature selection methods (Zhang et al., 2020b, 2023) that demonstrate promising performance when dealing with larger sample sets. These feature selection methods have the potential to be expanded and adapted for the selection of more complex MI-EEG feature representations.

Lastly, regarding feature adaptation, existing methods mainly focus on using maximum mean discrepancy (MMD) to express differences in feature distributions. However, this approach may have limitations in accurately characterizing the distribution of features, which can impact the adaptation process. In addition to using MMD to express distribution differences, researchers have explored alternative approaches such as domain-invariant kernel matrices (Ma et al., 2023) sparse coding (Chen and Song, 2021), and adversarial learning (Xu et al., 2023) to address this issue from different perspectives. To enhance the comprehensive representation of distribution differences, future researches should consider combining multiple measurement approaches to achieve better feature adaptation in cross-subject MI-EEG analysis.

Additionally, for the cross-subject MI-EEG classification, conventional methods (Zhang and Wu, 2020b; Luo, 2022) are limited by the design of classifiers and typically can only construct binary classifiers. For an MI-EEG dataset with *n* classes, either one class is selected as the target class, and the remaining total classes are considered as another class for binary classifier construction, or constructing n*(*n* − 1)/2 binary classification tasks. The advantage of DANN methods lies in their ability to handle classification tasks with any number of classes. However, they usually require a small number of samples for fine-tuning the DNN model, which can be less efficient in terms of calibration time compared to conventional methods. To address the dependency of conventional methods on binary classification, one approach is to construct multi-class classifiers such as kNN or SVM for the end-to-end multi-class domain adaptation (Zhou et al., 2023). Another approach involves incorporating the learned features into neural networks to achieve multi-class classification. Our proposed DS-KTL method defines the nearest class prototype using Equation 23. If the conditional probability calculation in Equation 24 is directly extended to multi-class scenario, it may lead to the clustering of multiple classes and deteriorate classification performance. Future works will focus on extending the pseudo-labels selection to deep neural network models by calculating the center loss (Zhang et al., 2020c) for different classes, replacing the nearest class prototype computed in Equation 23 to enable the classification of multi-classification.

Moreover, from the experimental results, we observed that in the STS strategy for the Dataset 2a (F vs. T) task, a higher classification accuracy was achieved when pseudo-labels selection was not used (see Figure 5B). This indicates that pseudo-labels selection has a negative impact on the feet vs. tongue task. In fact, previous studies (Pfurtscheller et al., 2006) have shown that the event-related desynchronization (ERD) phenomena in left hand and right hand MI are more distinct in the contralateral hemisphere of the brain, resulting in significant differences in sample distributions between the two classes (refer to Figure 4). Therefore, pseudo-labels selection can play a more significant role in such cases. However, for the feet and tongue MI, the ERD phenomena occur in the central region of the brain, where there is a higher degree of overlap. Although they are somewhat different, the overlap is substantial. Hence, for the feet vs. tongue classification task, during the computation of Equation 23, the nearest class prototypes of such two classes will overlap significantly. Consequently, during the pseudo-labels selection process, incorrect samples can be selected iteratively, leading to more severe performance degradation. To address this issue, future researches will refer to relevant works in metric learning (Wang et al., 2023a) to define novel class prototypes that better capture overlapping classes. By doing so, we can expect to achieve higher cross-subject MI-EEG classification performance.

## 6 Conclusion

MI-EEG classification plays an essential role in the development of BCI applications. However, due to the scarcity of subject-specific samples and the high cost of acquisition, cross-subject MI-EEG classification has become a prominent research focus. Feature adaptation is commonly employed to address this task. However, existing methods often overlook redundant feature dimensions and incorrectly predicted pseudo-labels, leading to performance bottlenecks in classification. In this paper, we propose the DS-KTL method to address these performance bottlenecks by simultaneously introducing feature selection and pseudo-label selection in the feature adaptation process. The proposed method first aligns samples through centroid alignment, followed by the extraction of RTS spatial features. The manifold embedded spatial feature selection method is then employed to select discriminative features for adaptation. In the feature adaptation stage, an efficient JPDA algorithm is introduced, along with three regularizations and pseudo-labels selection, to enhance the stability of JPDA iterations and ensure robust performance in cross-subject MI-EEG classification. Experimental evaluations conducted on two MI datasets using MTS/STS strategies demonstrate the effectiveness and efficiency of the DS-KTL method. The proposed method exhibits superior performance compared to SOTA methods, while achieving comparable efficiency. Ablation studies further confirm that the DS-KTL method ensures classification performance by selecting an appropriate number of features and parameter values. Future works will concentrate on exploring more effective feature representation of MI-EEG from a deep-learning perspective. Additionally, efforts will be directed toward developing more robust and efficient feature selection and adaptation methods. Furthermore, we aim to apply the DS-KTL method to practical BCI scenarios, such as emotional BCI and ERP-BCI.

## Data availability statement

The original contributions presented in the study are included in the article/supplementary material, further inquiries can be directed to the corresponding author.

## Author contributions

T-jL: Conceptualization, Data curation, Formal analysis, Funding acquisition, Investigation, Methodology, Project administration, Resources, Software, Supervision, Validation, Visualization, Writing—original draft, Writing—review & editing.
